# Future behavior of wind wave extremes due to climate change

**DOI:** 10.1038/s41598-021-86524-4

**Published:** 2021-04-12

**Authors:** Hector Lobeto, Melisa Menendez, Iñigo J. Losada

**Affiliations:** IHCantabria - Instituto de Hidráulica Ambiental de la Universidad de Cantabria, Santander, Spain

**Keywords:** Climate change, Projection and prediction, Ocean sciences, Civil engineering, Statistics

## Abstract

Extreme waves will undergo changes in the future when exposed to different climate change scenarios. These changes are evaluated through the analysis of significant wave height (H_s_) return values and are also compared with annual mean H_s_ projections. Hourly time series are analyzed through a seven-member ensemble of wave climate simulations and changes are estimated in H_s_ for return periods from 5 to 100 years by the end of the century under RCP4.5 and RCP8.5 scenarios. Despite the underlying uncertainty that characterizes extremes, we obtain robust changes in extreme H_s_ over more than approximately 25% of the ocean surface. The results obtained conclude that increases cover wider areas and are larger in magnitude than decreases for higher return periods. The Southern Ocean is the region where the most robust increase in extreme H_s_ is projected, showing local increases of over 2 m regardless the analyzed return period under RCP8.5 scenario. On the contrary, the tropical north Pacific shows the most robust decrease in extreme H_s_, with local decreases of over 1.5 m. Relevant divergences are found in several ocean regions between the projected behavior of mean and extreme wave conditions. For example, an increase in H_s_ return values and a decrease in annual mean H_s_ is found in the SE Indian, NW Atlantic and NE Pacific. Therefore, an extrapolation of the expected change in mean wave conditions to extremes in regions presenting such divergences should be adopted with caution, since it may lead to misinterpretation when used for the design of marine structures or in the evaluation of coastal flooding and erosion.

## Introduction

Changes in ocean wave climate, and especially in extremes, can have a significant effect on maritime activities such as seagoing shipping and the offshore industry. These changes combined together with those from other marine dynamics also affected by global warming (e.g. sea level rise^1^) may have a sensitive impact on coastal processes such as erosion^2–4^ and flooding^5–8^. Climate change can affect wave climate by the alteration of variables directly linked to its generation and propagation. Future variations in surface wind fields and marine ice coverage may alter wind-waves behavior over the oceans, changing the transmitted energy^9^ and generation fetch^10,11^.

Several studies have addressed the analysis of future changes in wave climate usually through wave climate projections. Since general circulation models do not simulate ocean wind-waves, wave climate projections are generated based on physical and atmospheric variable outputs from these general models (e.g. sea level pressure, surface wind fields, ice-coverage). Two are the main approaches used to generate wave climate projections both at regional and global scale: dynamical^12–17^ and statistical^18–20^. The COWCLIP community (Coordinated Ocean Wave Climate Project) is making remarkable efforts to integrate the existing global studies about future changes in wave climate and asses their robustness and uncertainties^21–24^. The results obtained in these studies evidence a consensus about the future increase in annual mean H_s_ in the Southern Ocean and tropical eastern Pacific, and a decrease in the North Atlantic and northwestern Pacific^25^.

In spite of its remarked relevance, the analysis of future changes in extreme wave climate under climate change scenarios deserves more attention. To this day, the assessment of the future variations in wave extremes has been carried out mainly through the analysis of high quantiles from wave height time series with a resolution higher than daily^26–29^ or annual maxima^12,16,20,27^. An accurate analysis of extreme events with a very low probability of occurrence needs to be grounded on the extreme value theory (EVT), which provides asymptotic long-term distributions for extremes and the approaches to estimate return values^30^. The limited number of studies on this topic^20,26,31–33^, commonly addressed through the assessment of changes in H_s_ return values, together with the intrinsic complexity of wave extremes, makes it difficult to reach a robust agreement about the regional projected changes by the end of the century. In order to better understand the future behavior of rare wave extremes, this study proposes the assessment of projected changes in H_s_ for different return periods under climate change scenarios, analyzing their magnitude, uncertainty, geographical distribution and their behavior with respect to mean wave conditions.

## Results

### Wave climate projections

Atmosphere–ocean general circulation models (GCMs) reproduce the intrinsic behavior of the real global climate, representing the extreme complexity inherent to the interaction between the atmosphere, the land surface, the ocean and the sea ice^34^. While GCMs faithfully reproduce the past global climate, in the projected period the behavior diverges from the historical trend due to the consideration of different future greenhouse gas (GHG) concentration scenarios. In this study, we run global dynamical wave simulations (see Methods) using sea surface winds and marine ice coverage outputs from GCMs to generate a multi-model wave climate ensemble consisting of seven members (further details on the GCMs characteristics and selection criteria in Supplementary Material). The resulting global wave projections (GWP) allow the study of hourly H_s_ time series at one-degree spatial resolution. We focus on the changes by the end of the century, comparing a climate reference historical period (1986–2005) to the future time slice 2081–2100 under RCP4.5 and RCP8.5 GHG emission scenarios. RCP4.5 and RCP8.5 are two of the representative concentration pathways covered in Fifth Assessment Report from IPCC (AR5)^35^, and represent GHG emission trajectories with a projected radiative forcing of 4.5 W m^−2^ and 8.5 W m^−2^ by 2100, respectively. The RCP8.5 scenario is representative of scenarios leading to high GHG levels whilst RCP4.5 comprises stabilization scenarios before 2100 by the employment of a range of adaptation technologies and strategies.

Although GCMs have the capability to reproduce the climate system and the interconnections between its components, certain issues such as the spatial resolution and the simplifications introduced by the numerical parametrizations of some physical processes cause a systematic bias inherent to each model^36,37^. Theses biases are transmitted to the wave propagations mainly due to the biases in sea surface wind fields^38^. In this context, different bias correction (BC) techniques have been successfully applied in several studies for different climatic variables such as precipitation^39–41^, temperature^40,42^ and wind^38,43^. More recently, BC has as well been applied to ocean wind-waves^44^, showing an unaltered change signal (i.e. the climate variability is preserved) and a possible decrease in the uncertainty of the projected changes. These precedents, together with the fact that extreme events are found to present the greatest biases^44^, make BC essential to assess stormy wave conditions. Thus, a correction technique is applied to reduce the biases of the GWP from each GCM (GWP_GCM_) in the reference period 1986–2005 and then extrapolate the correction to the future time slice. The global wave hindcast GOW2^45^ is used as reference historical data to conduct the BC due to its good performance for simulating extremes, especially in the tropical region.

**Figure 1:**
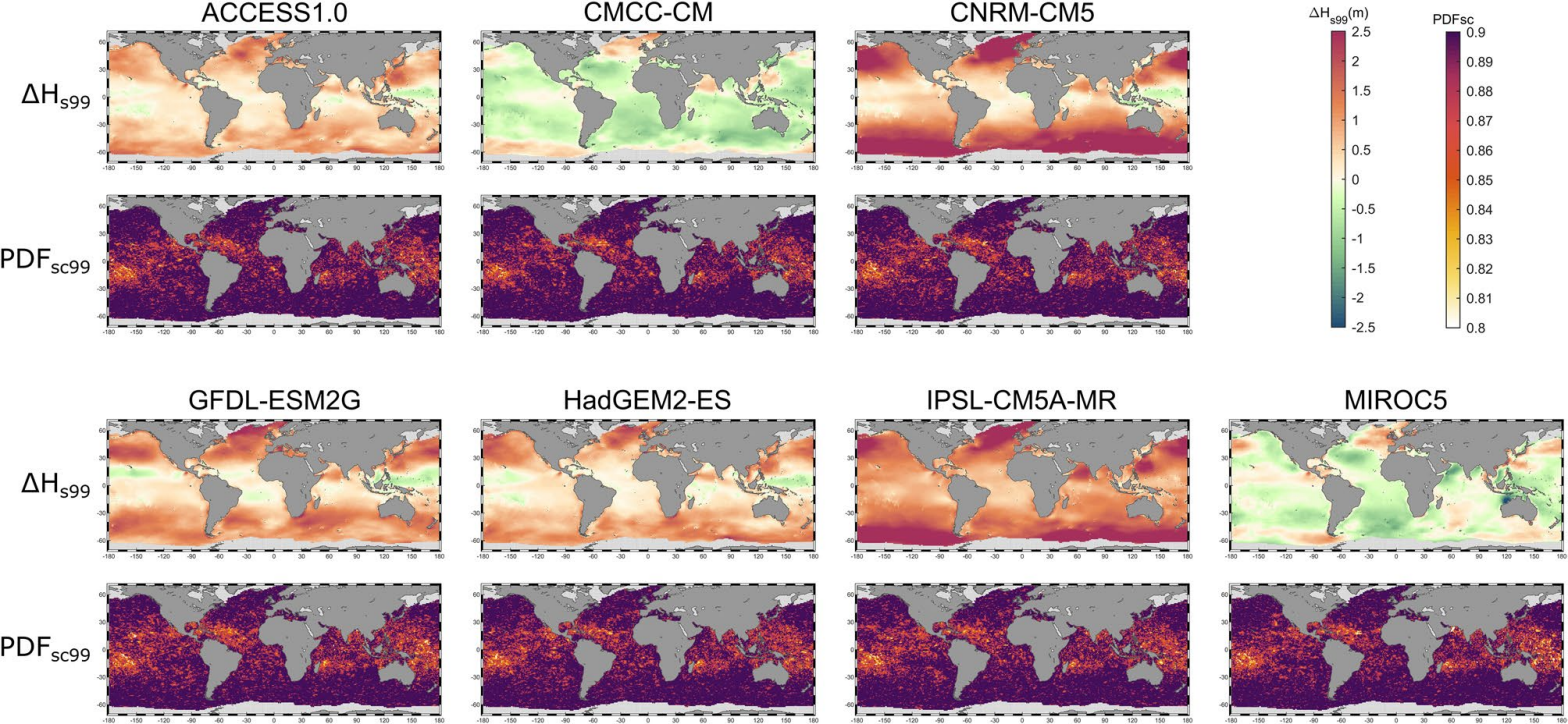
Upper panels: 99th percentile bias of significant wave height for each member of the ensemble (GWP_GCM_-GOW2). Lower panels: PDF-based skill score of the upper tail of the distribution over the 99th percentile after applying the bias correction under RCP8.5 scenario. Figure generated with MATLAB R2020a (https://es.mathworks.com/products/matlab.html).

The individual climate of each GCM varies at each run, precluding an hourly comparison between each GWP_GCM_ and the hindcast data. Hence, a bias correction method^44,46^ based on the adjustment of empirical quantiles is applied (see Methods). The 99th percentile bias indicates important differences between GCMs when compared with the reference historical data (Fig. 1). Thus, while GWP_CMCC_CM_ and GWP_MIROC5_ present the smallest discrepancies with GOW2 wave hindcast, GWP_IPSL_CM5A_MR_ and GWP_CNRM_CM5_ show the largest. In general, the differences are higher at latitudes above 35 degrees and lower in the tropical region. The performance of the BC is measured by comparing the probability density function (PDF) of H_s_ in the present and future time slices through the PDF-based skill score^47^ (PDF_sc_; see Methods). Results show a global effectiveness of the correction (Fig. 1) for every member of the ensemble, presenting values above 0.8 in almost all the global ocean. The lowest performance (i.e. white and yellow areas from PDF_sc_ maps in Fig. 1) is found in areas directly influenced by tropical cyclone activity, which evidence the difficulty of the BC to correct TC-induced wave extremes.

### Extreme value analysis

The behavior of wave extremes is studied through an extreme value analysis (EVA) of bias-corrected H_s_. In order to provide coherent results globally, a common method is applied in all regions notwithstanding the complexity involved in the different genesis of wave extremes. Considering this issue, we apply the generalized extreme value distribution (GEVD) parametric model based on the fit of annual maxima (AM) from hourly time series (see Methods). This is a widely used method in engineering together with exceedances over a threshold, although more mathematically stable for global studies and to analyze different climate simulations. Since the objective is to study the effect of climate change on extreme wave conditions using future time slices of 20 years, we consider a stationary statistical model assuming an unchanged behavior of the block maxima within these periods^48^. The regional distribution of the estimated location, scale and shape GEV parameters for the present-day wave climate and their future projected changes are discussed in Supplementary Material as a prelude of the future changes in the behavior of extreme wave events (Supplementary Fig. 3).

The low proportion of the global ocean showing both a statistically significant fit and a robust future change in the GEV shape parameter (14% and 16%, respectively) indicates an underlying instability when adding this degree of freedom in the extreme model to estimate return values, leading us to fit the AM to a type I EVD (also known as Gumbel distribution). The use of a simpler bi-parametric extreme model offers an overall greater robustness although it may lead to an underestimation of heavy-tailed distributions in tropical cyclone (TC) activity areas (Supplementary Fig. 3c). The goodness-of-fit (GoF) of the type I EVD is assessed with the Anderson–Darling (AD) test statistic^49^ (see Methods), a method proven to be optimal to analyze the performance of this distribution^50,51^ and widely applied in engineering studies to evaluate the fit of climatic extremes to extreme distributions^52–56^. Results show an overall good fit of the model except for some areas where the suitability of the Gumbel distribution is compromised (Supplementary Table 2 and Supplementary Fig. 4), mostly matching, as expected, the areas where the fit of the GEV shape parameter is found to be significant (Supplementary Fig. 3).

### Extreme wave climate future changes

**Figure 2:**
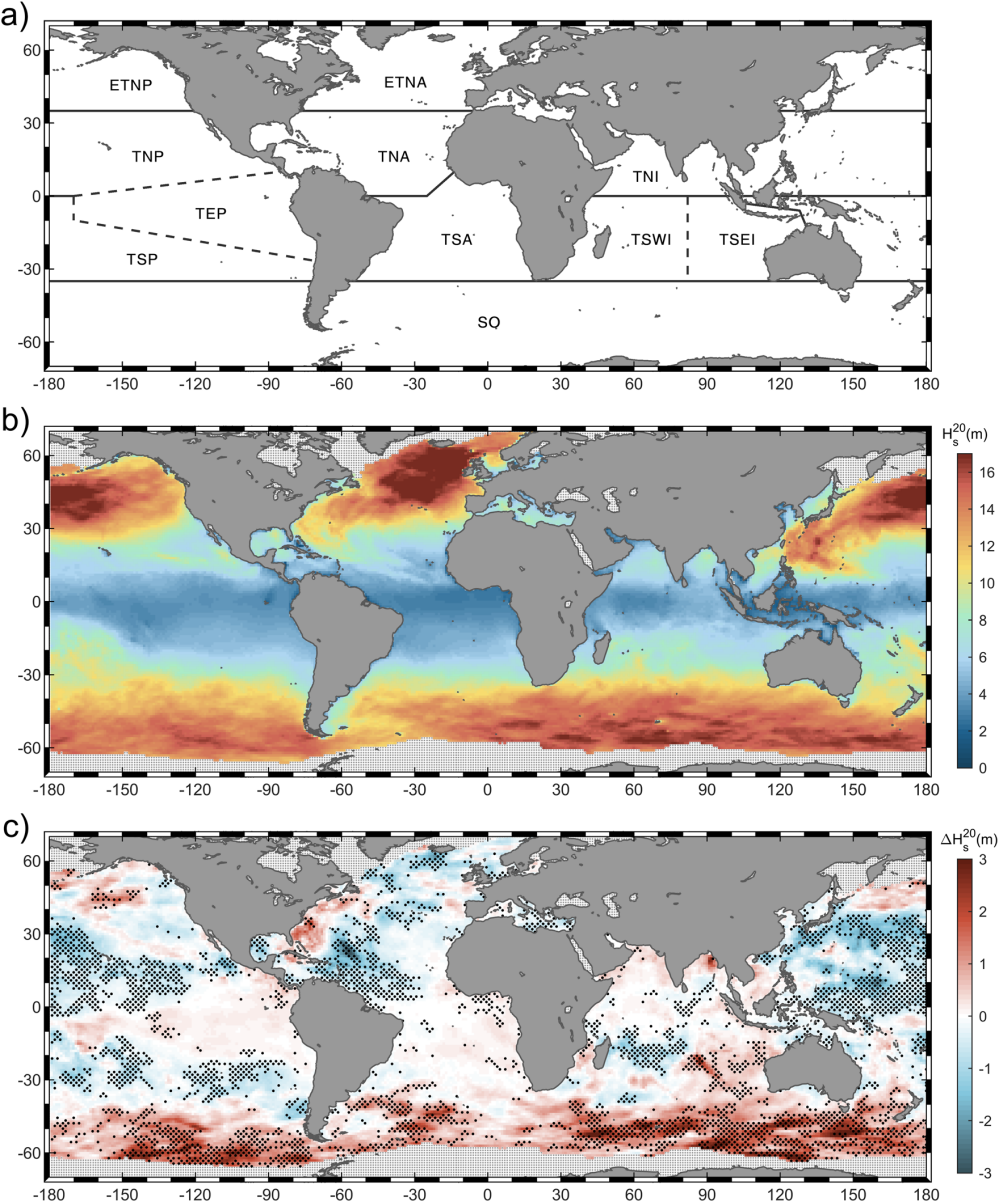
(**a**) Proposed global ocean regionalization. ETNP (extra-tropical north Pacific), ETNA (extra-tropical north Atlantic), TNP (tropical north Pacific), TNA (tropical north Atlantic), TNI (tropical north Indian), TEP (tropical eastern Pacific), TSP (tropical south Pacific), TSA (tropical south Atlantic), TSWI (tropical southwestern Indian), TSEI (tropical southeastern Indian), SO (Southern Ocean). (**b**) Present-day ${\mathrm{H}}_{\mathrm{s}}^{20}$ climate from GOW2 hindcast. (**c**) Multi-model ensemble mean change in ${\mathrm{H}}_{\mathrm{s}}^{20}$ (in meters) under RCP8.5 by the end of the century (2081–2100 relative to 1986–2005). Stippling denotes changes statistically significant at 95% confidence level in at least 50% of the members and an agreement in the sign of change in more than 80% of the members. Figure generated with MATLAB R2020a (https://es.mathworks.com/products/matlab.html).

Present-day wave climate estimates for very low probabilities of occurrence studied through the significant wave height parameter (5-, 20-, 50- and 100-year return periods, i.e. ${\mathrm{H}}_{\mathrm{s}}^{5}$, ${\mathrm{H}}_{\mathrm{s}}^{20}$, ${\mathrm{H}}_{\mathrm{s}}^{50}$ and ${\mathrm{H}}_{\mathrm{s}}^{100}$, respectively), evidence a very similar spatial pattern regardless the analyzed return level (Fig. 2b; Supplementary Fig. 7a–c). The increasing ${\mathrm{H}}_{\mathrm{s}}$ gradient from the equator to higher latitudes^57,58^ reaches its maximum in the northernmost regions of the Atlantic and Pacific oceans, showing magnitudes above 19 m and 22 m for ${\mathrm{H}}_{\mathrm{s}}^{20}$ and ${\mathrm{H}}_{\mathrm{s}}^{100}$, respectively. Similarly, the Southern Ocean is a highly energetic region that presents values up to 17 m and 20 m for ${\mathrm{H}}_{\mathrm{s}}^{20}$ and ${\mathrm{H}}_{\mathrm{s}}^{100}$ in the southern Indian Ocean. Note that the high magnitudes observed in the extra-tropical region extend to TC activity areas (e.g. tropical northeastern Atlantic, tropical northeastern Pacific or tropical southeastern Pacific), inducing a very strong gradient toward the low values found around the equator (i.e. magnitudes lower than 2.0 m for ${\mathrm{H}}_{\mathrm{s}}^{\mathrm{R}100}$). To provide confidence in the estimated H_s_ return values from model runs, a validation against return values estimated from buoy records is carried out. The buoy data set used to conduct the validation is obtained from the Global Ocean—Delayed Mode Wave Product from Copernicus Marine Service^59^. The selected buoys match a set of strict requirements to ensure a proper EVA and a reliable comparison with the outcomes of the numerical simulations developed at one-degree spatial resolution (see Methods). We finally define a set of fifty-two buoys located in the Atlantic and Pacific basins and both hemispheres (Supplementary Table 3). A mean relative error of 13%, 18% and 20% and a mean square error of 2 m, 7 m and 12 m are obtained after applying the bias correction for ${\mathrm{H}}_{\mathrm{s}}^{5}$, ${\mathrm{H}}_{\mathrm{s}}^{20}$ and ${\mathrm{H}}_{\mathrm{s}}^{50}$, respectively (Supplementary Fig. 6 and Supplementary Table 4).

The spatial distribution of the projected changes is also similar for different return periods although showing an increasing uncertainty for higher extreme estimates. A clear geographical pattern characterized by an overall expected increase in wave extremes in the Southern Ocean and a decrease in the Northern Hemisphere (NH) can be distinguished, presenting as well minor exceptions such as the northernmost Pacific and northwestern Atlantic. The tropical south region, however, shows a heterogeneous change pattern in which both negative and positive trends can be observed. Hence, a regionalization of the global ocean is proposed to better understand the future changes under climate change scenarios and their uncertainty (Fig. 2a and further details in Supplementary Material). Table 1 summarizes the proportion of area in which changes are found to be robust and the average projected change for all the analyzed return periods in each region under RCP4.5 and RCP8.5 scenarios. The uncertainty assessment of the projected changes is fully described in Methods.

**Table 1:** For each analyzed ocean region, upper row: proportion of the region showing robust changes and (between brackets) proportion of the region showing a robust increase.

	RCP4.5				RCP8.5			
	${\mathrm{H}}_{\mathrm{s}}^{5}$	${\mathrm{H}}_{\mathrm{s}}^{20}$	${\mathrm{H}}_{\mathrm{s}}^{50}$	${\mathrm{H}}_{\mathrm{s}}^{100}$	${\mathrm{H}}_{\mathrm{s}}^{5}$	${\mathrm{H}}_{\mathrm{s}}^{20}$	${\mathrm{H}}_{\mathrm{s}}^{50}$	${\mathrm{H}}_{\mathrm{s}}^{100}$
**Global**								
Robustness (%)	22.4 _(10.5)_	18.2 _(8.7)_	16.8 _(8.1)_	16.2 _(7.7)_	**31.6 **_**(14.2)**_	**27.6 **_**(12.3)**_	**25.4 **_**(11.3)**_	24.3 _(10.8)_
Avg. change (m)	+ 0.0 _(+0.0; +0.8)_	+ 0.1 _(+0.0; +1.1)_	+ 0.1 _(+0.0; +1.3)_	+ 0.1 _(+0.0; +1.5)_	+ **0.1 **_**(+0.0; +1.5)**_	+ **0.1 **_**(+0.0; +1.8)**_	+ **0.1 **_**(+0.0; +2.0)**_	+ 0.1 _(+0.0; +2.2)_
**ETNP**								
Robustness (%)	16.3 _(8.5)_	12.4 _(4.8)_	12.2 _(4.9)_	11.9 _(4.5)_	15.6 _(5.9)_	12.6 _(5.5)_	11.4 _(5.2)_	11.6 _(5.1)_
Avg. change (m)	+ 0.1 _(+0.0; +0.7)_	+ 0.1 _(+0.0; +1.1)_	+ 0.1 _(+0.0; +1.3)_	+ 0.2 _(+0.0; +1.5)_	+ 0.0 _(+0.0; +0.8)_	+ 0.0 _(+0.0; +1.2)_	+ 0.0 _(+0.0; +1.5)_	+ 0.0 _(+0.0; +1.7)_
**TNP**								
Robustness (%)	**31.1 **_**(0.7)**_	22.2 _(1.1)_	19.9 _(1.5)_	18.6 _(1.9)_	**44.6 **_**(0.1)**_	**42.4**_**(0.3)**_	**39.6 **_**(0.3)**_	**38.1 **_**(0.4)**_
Avg. change (m)	− **0.2 **_**(−0.0; −0.9)**_	− 0.2 _(**−**0.0; **−**1.3)_	− 0.2 _(**−**0.0; **−**1.5)_	− 0.2 _(**−**0.0; **−**1.7)_	− **0.5 **_**(−0.1; −1.2)**_	− **0.5 **_**(−0.1; −1.5)**_	− **0.6 **_**(−0.1; −1.7)**_	− **0.6 **_**(−0.1; −1.9)**_
**TEP**								
Robustness (%)	11.1 _(9.2)_	8.3 _(6.8)_	7.3 _(5.7)_	7.1 _(5.3)_	9.4 _(8.1)_	9.1 _(6.1)_	8.7 _(5.3)_	8.6 _(5.2)_
Avg. change (m)	+ 0.0 _(+0.0; +0.1)_	+ 0.0 _(+0.0; +0.2)_	+ 0.0 _(+0.0; +0.3)_	+ 0.0 _(+0.0; +0.3)_	+ 0.1 (0.0;+ 0.3)	+ 0.1 _(+0.0; +0.4)_	+ 0.1 _(+0.0; +0.4)_	+ 0.1 _(+0.0; +0.5)_
**TSP**								
Robustness (%)	24.0 _(1.0)_	20.7 _(1.0)_	19.8 _(1.3)_	19.5 _(1.6)_	**30.6 **_**(0.2)**_	**27.2 **_**(0.5)**_	**25.9 **_**(0.8)**_	**25.5 **_**(0.7)**_
Avg. change (m)	− 0.2 _(**−**0.0; **−**0.6)_	− 0.2 _(**−**0.0; **−**0.9)_	− 0.2 _(**−**0.0; **−**1.1)_	− 0.2 _(**−**0.0; **−**1.2)_	− **0.2 **_**(−0.0; −0.6)**_	− **0.3 **_**(−0.0; −0.9)**_	− **0.3 **_**(−0.0; −1.1)**_	− **0.4 **_**(−0.1; −1.2)**_
**ETNA**								
Robustness (%)	18.4 _(1.7)_	13.4 _(2.2)_	11.7 _(2.1)_	10.7 _(1.9)_	**25.3 **_**(1.6)**_	17.6 _(2.1)_	14.7 _(2.4)_	12.8 _(2.2)_
Avg. change (m)	− 0.3 _(**−**0.0; **−**0.9)_	− 0.2 _(**−**0.0; **−**1.1)_	− 0.2 _(**−**0.0; **−**1.2)_	− 0.2 _(**−**0.1; **−**1.4)_	− **0.3 **_**(−0.1; −1.0)**_	− 0.3 _(**−**0.0; **−**1.2)_	− 0.3 _(**−**0.1; **−**1.4)_	− 0.2 _(**−**0.0; **−**1.5)_
**TNA**								
Robustness (%)	**25.0 **_**(2.5)**_	22.2 _(2.9)_	20.7 _(3.0)_	21.0 _(2.9)_	**37.7 **_**(0.6)**_	**31.4 **_**(1.0)**_	**29.3 **_**(0.9)**_	**28.5 **_**(0.8)**_
Avg. change (m)	− **0.1 **_**(−0.0; −0.4)**_	− 0.0 _(**−**0.0; **−**0.6)_	− 0.0 _(**−**0.0; **−**0.8)_	− 0.0 _(**−**0.0; **−**0.8)_	− **0.3 **_**(−0.0; −1.0)**_	− **0.4 **_**(−0.0; −1.4)**_	− **0.4 **_**(−0.0; −1.7)**_	− **0.4 **_**(−0.0; −2.0)**_
**TSA**								
Robustness (%)	15.4 _(13.2)_	15.5 _(12.4)_	15.2 _(11.7)_	14.6 _(11.2)_	10.2 _(8.3)_	10.4 _(8.4)_	10.2 _(8.1)_	10.1 _(8.1)_
Avg. change (m)	+ 0.0 _(+0.0; +0.2)_	+ 0.1 _(+0.0; +0.4)_	+ 0.1 _(+0.0; +0.5)_	+ 0.1 _(+0.0; +0.5)_	+ 0.0 _(+0.0; +0.2)_	+ 0.1 _(+0.0; +0.3)_	+ 0.1 _(+0.0; +0.4)_	+ 0.1 _(+0.0; +0.4)_
**TNI**								
Robustness (%)	12.5 _(3.4)_	11.3 _(5.8)_	10.6 _(5.9)_	11.3 _(6.9)_	15.8 _(3.4)_	10.7 _(4.2)_	8.8 _(4.1)_	8.5 _(4.6)_
Avg. change (m)	+ 0.0 _(+0.0; +0.3)_	+ 0.0 _(+0.0; +0.4)_	+ 0.1 _(+0.0; +0.5)_	+ 0.1 _(+0.0; +0.6)_	+ 0.0 _(+0.0; +0.5)_	+ 0.1 _(+0.0; +0.7)_	+ 0.2 _(+0.0; +0.8)_	+ 0.2 _(+0.0; +0.9)_
**TSWI**								
Robustness (%)	15.1 _(4.8)_	16.3 _(7.2)_	15.8 _(6.8)_	15.9 _(6.9)_	24.3 _(2.0)_	21.9 _(2.5)_	20.5 _(2.7)_	20.1 _(3.2)_
Avg. change (m)	− 0.0 _(**−**0.0; **−**0.5)_	− 0.0 _(**−**0.0; **−**0.8)_	− 0.0 _(**−**0.0; **−**0.9)_	− 0.1 _(**−**0.0; **−**1.1)_	− 0.1 _(**−**0.0; **−**0.6)_	− 0.1 _(**−**0.0; **−**1.0)_	− 0.2 _(**−**0.0; **−**1.2)_	− 0.2 _(**−**0.0; **−**1.3)_
**TSEI**								
Robustness (%)	19.7 _(15.4)_	18.8 _(14.0)_	19.2 _(14.3)_	19.1 _(14.0)_	20.6 _(14.6)_	**27.3 **_**(21.0)**_	**27.6 **_**(22.0)**_	**28.4 **_**(22.9)**_
Avg. change (m)	+ 0.1 _(+0.0; +0.6)_	+ 0.2 _(+0.0; +0.9)_	+ 0.3 _(+0.0; +1.2)_	+ 0.3 _(+0.0; +1.3)_	+ 0.1 _(+0.0; +0.5)_	+ **0.2 **_**(+0.0; +0.9)**_	+ **0.3 **_**(+0.1; +1.1)**_	+ **0.4 **_**(+0.1; +1.3)**_
**SO**								
Robustness (%)	**25.3 **_**(23.6)**_	20.2 _(18.2)_	18.2 _(16.2)_	17.1 _(15.0)_	**41.4 **_**(39.7)**_	**34.3 **_**(32.5)**_	**30.9 **_**(29.2)**_	**28.8 **_**(27.0)**_
Avg. change (m)	+ **0.3 **_**(+0.0; +1.0)**_	+ 0.4 _(+0.1; +1.3)_	+ 0.5 _(+0.1; +1.5)_	+ 0.5 _(+0.1; +1.7)_	+ **0.6 **_**(+0.1; +1.7)**_	+ **0.8 **_**(+0.1; +2.2)**_	+ **0.9 **_**(+0.1; +2.5)**_	+ **0.9 **_**(+0.1; +2.8)**_

Lower row: average magnitude of the projected changes and (between brackets) 5th and 95th percentiles of the projected changes with the same sign as the estimated average in the region. Results are displayed for 5-, 20-, 50- and 100-year return periods under RCP4.5 and RCP8.5 scenarios. Changes characterized by a proportion higher than 25% are highlighted in bold font. All results are rounded to one decimal place.

To facilitate the understanding of the results, in the following we will use the projected changes in ${\mathrm{H}}_{\mathrm{s}}^{20}$ as the baseline of the exposition throughout the text. Results for the rest of return periods are shown and discussed in Supplementary Material. The estimated future changes in ${\mathrm{H}}_{\mathrm{s}}^{20}$ under RCP8.5 scenario (Fig. 2c; Table 1) exhibits a robust increase in a 34% of the Southern Ocean with a higher uncertainty in the Atlantic region (from 60°W to 30°E). The agreement between this increasing pattern and the projected changes found in previous studies^20,31–33^ based on different scenarios, models and EVA approaches, provide confidence to the expected behavior of wave extremes in SO. The proportion of robust changes increases with latitude, reaching 29% in the roaring forties (between 40°S and 50°S) and 45% in the furious fifties (between 50°S and 60°S) regions. The average expected change of + 0.8 m, with local increases of over 3.5 m, makes SO the region where the greatest change in extreme H_s_ is expected. Furthermore, this projected increase can also be interpreted as an increase by 2100 in the frequency of occurrence of waves associated at present to a 20-year return period, i.e. present-day ${\mathrm{H}}_{\mathrm{s}}^{20}$ would have associated by 2100 a return period lower than 20 years. In this regard, almost half SO region (42%) shows a return period of present-day ${\mathrm{H}}_{\mathrm{s}}^{20}$ lower than 10 years by the end of the century, with local areas presenting return periods lower than 5 years (Supplementary Fig. 8b). The effect of the intensification and poleward shift of the southern extra-tropical storm track^60,61^ has been already discussed in previous wave climate studies^23,33^ as a plausible cause of the expected increase in H_s_. In addition, the projected increase in the frequency of extreme extra-tropical cyclones in the Southern Hemisphere (SH) found from the analysis of CMIP5 ensembles^61,62^, might as well be related to the obtained change. In particular, the latter is especially significant between 45°S and 60°S and in the Southern Indian Ocean, which is consistent with the found spatial pattern.

Regarding the extra-tropical north region, the Pacific (13%) and Atlantic (18%) basins show a spatial change pattern characterized by the presence of small robust areas dispersedly distributed that induce an average change with opposite sign (+ 0.0 m and − 0.3 m, respectively). These results could be explained by the great uncertainty associated to the effect of global warming on northern extratropical storm tracks^25^, the main forcing of these events, due to great discrepancies between models^63^. In addition, the good fit of the extreme model above 35°N shown by AD test statistic (Supplementary Fig. 4), supports the model definition of storm tracks as the main source of uncertainty of the estimated changes. Notwithstanding these limitations, the poleward shift in the Pacific storm track shown in previous studies ^64,65^ and the ice cover reduction are the most likely causes of the positive average change found in ETNP.

The assessment of tropical projected changes needs to be done cautiously. Results in regions affected by tropical cyclone activity are directly conditioned by the skill of the models to reproduce these storms and the capability of the extreme distribution to model their behavior. The former is surely influenced by the spatial resolution of the models (in this case from 0.75° to 2.5°), although other features such as the physical parametrizations and dynamical cores should also be considered^66,67^. The skill of the models is analyzed by comparing the maximum wave height registered by the ensemble members and the reference hindcast as has been similarly done in previous studies^66,68^. Results show that although all the models simulate TCs, there is a general underestimation of the maximum wave height and sensitive differences between members (see Supplementary Material). Nevertheless, the applied bias correction helps to correct the magnitude in these areas, providing more accurate results. Concerning the model performance, the AD test statistic evidences a poor fit of TC-induced extremes to the Gumbel distribution in these regions as expected from the analysis of the GEV shape parameter (Supplementary Fig. 2c).

In spite of these issues, the Pacific Ocean shows a consistent tropical decrease except for the eastern part, where a highly uncertain increase can be observed (< 10% of TEP show robust changes). TNP shows a negative change (42%; − 0.5 m) consistent with the projected decrease in low-to-mid latitude winds^12^ in the Pacific most likely caused by the poleward shift expected for the northern storm track^15^, becoming the region with the highest robustness among the eleven analyzed. This projected decrease implies a correlative increase in the return period of present-day ${\mathrm{H}}_{\mathrm{s}}^{20}$ by the end of the century, which exceeds 50 years in a notable proportion of the region (45%) (Supplementary Fig. 8b). Similarly, the strong decrease found in TSP (27%; − 0.3 m) may be as well explained by the poleward displacement of the southern storm track. Nevertheless, note that the westernmost part of both regions, the areas most affected by intense TCs in the models (see supplementary Fig. 5), show great uncertainty and a heterogenous change pattern, precluding a consistent conclusion about the projected changes. TNA (31%) is mostly characterized by a decreasing pattern with an average change of − 0.4 m, which agrees with the robust decrease found by Meucci et al. for 100-yr return period H_s_^31^. The low ability of the models to reproduce the TC activity areas (see supplementary Fig. 5 for GOW2 hindcast) makes it very difficult to reach a consistent conclusion about the cause of the obtained projected changes. For example, the projected decrease found in the tropical northwestern Atlantic may be caused by the decrease in low-intense TCs or, more likely, by a westward extension of the negative change induced by the projected decrease in northern mid-to-low latitude winds^12,15^ observed between 30°W and 50°W. Consistently with the Pacific basin, the westernmost part of the region affected by intense TCs shows great uncertainty in the projected changes. However, a homogenous increase can be observed around Florida that agrees with the expected increase in wind speed of major TCs in the Atlantic Ocean^69^. In the tropical south region, however, a weak increase in extreme waves is obtained (10%; + 0.1 m). The change pattern in the tropical Indian Ocean is characterized by a bipolar behavior in the southern region so that while a decrease is expected on the west (22%; − 0.1 m), the sign of change on the east turns out to be the opposite (27%; + 0.2 m). The latter implies that the extreme waves that at present reach the western coast of Australia on average once every 20 years, by 2100 would do it with a return period lower than 15 years (Supplementary Fig. 8b). Contrarily to the Pacific and Atlantic basins, the tropical north Indian Ocean shows great uncertainty (11%).

Projected changes in ${\mathrm{H}}_{\mathrm{s}}^{20}$ under RCP4.5 scenario (Supplementary Fig. 9b; Table 1) show an overall great similarity in the spatial change pattern (same sign of change in every region) and higher uncertainty (proportion of the global ocean with a robust projected change reduces from 28 to 18%) with respect to RCP8.5. The most notable variations in the magnitude of the average change are observed in SO (from + 0.8 to + 0.4 m), TNP (from − 0.5 to − 0.2 m) and TNA (from − 0.4 to − 0.0 m) regions. Note that the latter case is caused by the projected increase found around Florida, which far from reducing the robustness and magnitude of the changes found for RCP8.5 scenario, show an increase in both features regardless the analyzed return level.

**Figure 3:**
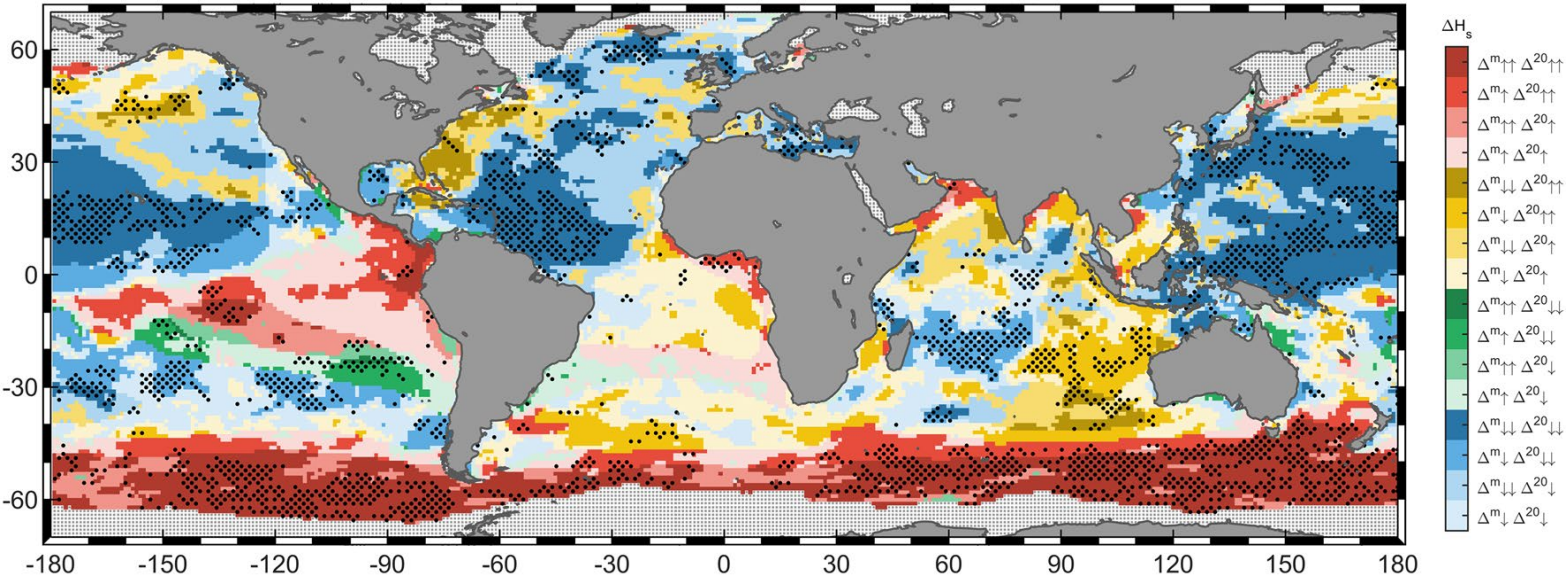
Comparison of projected changes in mean (${\Delta }^{\mathrm{m}}$) and extreme (${\Delta }^{20}$) significant wave height under RCP8.5 scenario by the end of the century (2081–2100 relative to 1986–2005). Colors represent the combinations of sign of change between mean and extreme wave conditions: red indicates an increase in both conditions, blue represent a decrease in both conditions, yellow represent a decrease in mean and an increase in extreme conditions and green represents an increase in mean and a decrease in extreme conditions. For each combination, one arrow indicates a relative change lower than the global median and two arrows indicate relative changes higher than the global median. The global median is obtained as the median of the relative changes with the same sign as the analyzed variation. Stippling denotes changes statistically significant at 95% confidence level in at least 50% of the members and an agreement in the sign of change in more than 80% of the members for both mean and extreme significant wave height. Figure generated with MATLAB R2020a (https://es.mathworks.com/products/matlab.html).

Despite the future changes in mean wave height have been assessed in several studies ^14,22,23^, the differences between the expected behavior of mean and extreme wave conditions with a low occurrence rate under climate change scenarios is still unclear. In this regard, it is essential to determine whether future changes in wave extremes have the same spatial pattern as those from mean conditions, likely indicating a shift of the probability distribution that is affecting the tails in a similar way, or if there exist other causes that may be the source of the discrepancies between both behaviors (e.g. specific changes in intense circulation patterns where internal feedbacks of the climate system are the main drivers). Therefore, in order to evaluate the similarities and differences between the future behavior of mean and extreme wave conditions, we compare the projected relative changes in annual mean (${\mathrm{H}}_{\mathrm{s}}^{\mathrm{m}}$) and 20-year return period (${\mathrm{H}}_{\mathrm{s}}^{20}$) significant wave height under RCP8.5 (Fig. 3) and RCP4.5 (Supplementary Fig. 10) scenarios. We assess the agreement in the sign of change between changes in mean and extreme H_s_ (blue, green, yellow and red colors of Fig. 3), ranking variations as low or large by comparison with the global median (one or two arrows in Fig. 3). The combined uncertainty of the changes is also analyzed (stippling denotes robust changes in ${\mathrm{H}}_{\mathrm{s}}^{\mathrm{m}}$ and ${\mathrm{H}}_{\mathrm{s}}^{20}$).

Although an overall agreement in the sign of change under RCP8.5 scenario is found (blue and red in Fig. 3), relevant differences in the spatial change pattern (yellow and green in Fig. 3) and in the uncertainty assessment can also be observed. There is a general expected decrease in ${\mathrm{H}}_{\mathrm{s}}^{\mathrm{m}}$ in the whole NH (Supplementary Fig. 11a), being the North Atlantic and northwestern Pacific the regions that, in line with the existent consensus^22,23,25^, present the most robust projected changes. This robust decreasing uniformity found for mean conditions is not met for extremes since certain areas located in the Atlantic (e.g. east coast of North America), Pacific (e.g. northeastern region) and Indian oceans show the opposite sign of change (i.e. increase), accompanied, in many cases, by a remarkable increase in the uncertainty. Among all these areas of disagreement in the sign of change, the northeastern Pacific stands out due to the robustness and the high magnitude of the obtained changes (darkest yellow colors in Fig. 3). In addition, it is especially noteworthy the great uncertainty and heterogenous pattern found in the northernmost Atlantic for extremes, likely corroborating a different cause for the expected changes than only a direct shift of the distribution.

Concerning the SH, projected changes in ${\mathrm{H}}_{\mathrm{s}}^{\mathrm{m}}$ are robust in a high percentage of the region (Supplementary Fig. 11a), which is consistent with previous studies about the expected increase in the Southern Ocean and tropical eastern Pacific^22,23,25^. Changes in ${\mathrm{H}}_{\mathrm{s}}^{20}$ are also robust in a great percentage of the Southern Ocean, therefore leading to a strong agreement between extreme and mean conditions. Although a high percentage of the south tropical region shows robust projected changes in ${\mathrm{H}}_{\mathrm{s}}^{\mathrm{m}}$ (60% from 35 degrees south to the equator), the great uncertainty related to extremes causes that only around 13% of the whole region shows robust changes in both conditions. In spite of this, regions such as the tropical south Indian Ocean presents robust future changes in mean and extreme wave height, projecting a decrease in the whole region in ${\mathrm{H}}_{\mathrm{s}}^{\mathrm{m}}$ and a bipolar change in ${\mathrm{H}}_{\mathrm{s}}^{20}$ characterized by an increase in the eastern part consistent with previous studies^31^. Note that this strong disagreement is observed in the very same area where a robust increase in the GEV scale parameter is found (Supplementary Fig. 3e). Finally, it is also remarkable how the same wedge-shaped increasing pattern likely caused by the expected intensification of southeasterly trades^23^, appears in the tropical eastern Pacific Ocean for mean and extreme conditions. Nevertheless, a severe increase in the uncertainty for extremes and an amplification for mean waves denoted by the presence of green areas in the southern part can be observed.

The increase in the uncertainty previously seen for changes in H_s_ return values under RCP4.5 scenario with respect to RCP8.5 also exists for future variations in ${\mathrm{H}}_{\mathrm{s}}^{\mathrm{m}}$ (Supplementary Fig. 11b), reducing the proportion of the global ocean that shows robust changes from 63 to 46%. Hence, this inevitably involves a decrease in the proportion of area in which changes are consistent for ${\mathrm{H}}_{\mathrm{s}}^{\mathrm{m}}$ and ${\mathrm{H}}_{\mathrm{s}}^{20}$ (from 19 to 9%; Supplementary Fig. 10). Nonetheless, the agreement in the sign of change between mean and extreme H_s_ show a pretty similar spatial pattern as for the RCP8.5 scenario. Most remarkable differences are found in the northeastern Pacific and westernmost north Atlantic, where an amplification of the area showing a projected increase in ${\mathrm{H}}_{\mathrm{s}}^{20}$ and a decrease in ${\mathrm{H}}_{\mathrm{s}}^{\mathrm{m}}$ can be observed.

## Concluding remarks

There is a consensus among the climate community about the future changes in annual mean H_s_ due to climate change by the end of the century in several regions around the global ocean^21,22,25^. Nonetheless, the future behavior of extreme events with a very low probability of occurrence (i.e. return values) and the similarities and differences with respect to the expected for mean wave conditions are yet to be fully explored. In order to assess projected changes in wind-wave extremes, we apply a statistical extreme analysis commonly used in engineering to calculate design return periods. The outcomes evidence that the type I extreme value distribution can be used from hourly time series to obtain robust statistics in almost all the global ocean. Only areas directly influenced by tropical cyclone activity may underestimate extreme wave heights (Supplementary Fig. 3c) as they are characterized by heavy-tailed distributions.

In this study, we assess the projected changes in H_s_ extreme events for 5-, 20-, 50- and 100-year return periods. The obtained changes and the proposed regionalization (Fig. 2a) open the possibility of reaching consistent conclusions on the future regional variability of wave extremes and comparing them with the projected changes in mean wave heights. In this regard, although an overall spatial concordance in the sign of change is observed (approximately two thirds of the global ocean; Fig. 3 and Supplementary Fig. 10), robust discrepancies also emerge in some areas by the end of the century concerning not only this feature, but also the magnitude and uncertainty. Hence, inferring the behavior of wave extremes from projected changes in mean conditions may lead to misinterpretation when used for the assessment of coastal processes or in the design of maritime infrastructures in these areas (e.g. sediment transport depends on ${\left({\mathrm{H}}_{\mathrm{s}}\right)}^{2}$; breakwater armor unit weight depends on ${\left({\mathrm{H}}_{\mathrm{s}}\right)}^{3}$, design loads of offshore structures depend on ${\mathrm{H}}_{\mathrm{s}}^{100}$). The possible origin of the projected changes in return values is as well discussed throughout the text, although specific regional analyses are recommended to discern the actual causes and their different contribution.

In addition to the increasing uncertainty found for changes in less frequent extremes (e.g. robustness of changes at global scale reduces from 32% for ${\mathrm{H}}_{\mathrm{s}}^{5}$ to 24% for ${\mathrm{H}}_{\mathrm{s}}^{100}$ under RCP8.5 scenario), two general conclusions can be drawn as return periods are higher. First, the proportion of positive changes increases, leading to an increase in the global average change (from − 0.5% for ${\mathrm{H}}_{\mathrm{s}}^{5}$ to + 0.3% for ${\mathrm{H}}_{\mathrm{s}}^{100}$ and from − 0.0% to + 0.6% for RCP8.5 and RCP4.5, respectively). Second, the magnitude of the observed changes is higher for regions with positive changes.

The Southern Ocean exhibits a consistent increase in H_s_ return values characterized by an average change up to + 0.5 m for RCP4.5 and + 0.9 m for RCP8.5 scenarios, showing for the latter local increases larger than 3.5 m for 20-year return period and higher. In the North Atlantic Ocean, although a robust homogenous decrease can be observed for ${\mathrm{H}}_{\mathrm{s}}^{\mathrm{m}}$, changes for extremes are characterized by great uncertainty with the sole exception of the decrease found in the tropical northwestern Atlantic. In addition, a low-robust increase is obtained in the westernmost part of the basin (USA and Canadian coasts) and some other European areas. The Pacific Ocean shows a robust general decrease in extreme events in the tropical region. This decreasing signal, however, is not found in the tropical eastern Pacific (the genesis area of the ENSO phenomenon), where projections provide a robust increase in mean conditions and an uncertain rise for extremes. The Indian Ocean shows remarkable differences between projected changes in mean and extreme H_s_. These discrepancies are especially relevant in the tropical southeastern region along the west Australian and Indonesian coasts, where the results indicate a robust increase in H_s_ return values and a decrease in ${\mathrm{H}}_{\mathrm{s}}^{\mathrm{m}}$.

Changes in extreme events are smaller and less robust for lower emission scenarios. The robustness of the projected changes reduces from RCP8.5 to RCP4.5 globally around 10% for any of the estimated return periods (e.g. from 25 to 17% for 50-year return period). This fact indicates that future impacts due to both extreme and mean wave height will be conditioned by the strategies taken for reducing greenhouse gas emissions.

Projected changes in areas affected by TC activity should be considered carefully as different issues preclude consistent conclusions. First, to this day and in spite of the important efforts done to elucidate the effect of global warming on tropical cyclones, there is still no consensus about the expected changes in frequency, intensity and tracks^69^. Second, the global resolution of the selected models^67^ (i.e. from 0.75° to 2.5°; see Supplementary Material), among other factors, induce their limited skill in reproducing these storms, which leads to a general underestimation of extreme waves generated by TCs (Supplementary Fig. 5). Finally, the goodness-of-fit analysis show a low-robust fit of the type I EVD in TC activity areas (Supplementary Fig. 4) as they are mostly characterized by heavy-tailed distributions. The above mentioned limitations in addressing this issue have also been observed in previous studies at global scale^20,31^, evidencing the need for progress in the field of climate projections on TCs, as well as the development of a specific approach to assess the future changes in TC-induced wave climate to obtain more accurate results^66,68^. For the latter, a regional approach based on synthetic TCs may be a suitable option. In fact, it has already been done for other sea surface dynamics (storm surge) for the present climate around the coastline of Australia^70^ or, more recently, for climate change scenarios along US Atlantic and Gulf Coasts^71^.

Future changes in the behavior of wave extremes may have severe implications on maritime shipping and the offshore industry (e.g. gas and oil fields). Although this analysis is out of the scope of the present study, the projected changes obtained here could help to unravel the impact that these variations may have on the said economic activities. In order to contribute to further research in this matter, the global projected changes at one-degree spatial resolution for the two emission scenarios and all the analyzed return periods will be openly shared online. Similarly, the changes in extreme wave height due to climate change may have an important impact in coastal processes due for instance to its direct implication in composing extreme sea levels or in sediment transport. Nevertheless, it is important to highlight that the main goal of this study is to describe how the behavior of events with a very low probability of occurrence will change across the ocean basins, not addressing how to transfer those changes to the coast. Therefore, a downscaling procedure would be required to develop an accurate analysis on the projected changes in wave extremes nearshore.

## Methods

### Bias correction (BC)

The empirical quantile mapping technique^46^ (EQM), slightly modified to better correct the upper-tail of the distribution, is the method selected to correct biases from GWP_GCM_. EQM consists in adjusting the empirical cumulative distribution function (CDF) from each GWP_GCM_ to the empirical CDF from the reference historical data (Eq. 1). Thus, a correcting increment ($${X}_{corr})$$ is obtained for each selected quantile and then a linear interpolation is applied between them. The correction outside the range defined by the selected quantiles is the same as the applied to the first or the last quantiles.

1 $${X}_{corr}={CDF}_{Ref}^{-1}\left[{CDF}_{GCM}(X)\right]$$

where ${CDF}_{Ref}$ is the cumulative distribution function of the reference data and ${CDF}_{GCM}$ is the cumulative distribution function of each ${GWP}_{GCM}$.

The quantile selection plays an important role in the adequate performance of the method. The selected quantile probabilities (${q}_{i}$) are defined as follows:

- Quantile 0.01 (${q}_{01}$) followed by linearly distributed quantiles from 0.05 (${q}_{05}$) to 0.90 (${q}_{90}$) each 0.05.
- Twelve Gumbel scaled quantiles (Eq. 2) from ${q}_{90}$ for a better representation of the upper tail of the distribution.
  2 $${q}_{i}=exp\left[-exp\left(-{x}_{qi}\right)\right]$$

The same correction is applied to the 20-year time-slice at the end of the century (2081–2100).

The performance of the bias correction is evaluated through the PDF-based skill score (${PDF}_{sc}$)^47^. It is based on the comparison between the probability density function (PDF) of the reference data and the GWP_GCM_ for the present-day climate (Eq. 3).

3 $${PDF}_{sc}=\sum_{i=1}^{n}\mathrm{min}\left(f\left({H}_{s,i}^{Ref}\right), f\left({H}_{s,i}^{GCM}\right)\right)\cdot \Delta {H}_{s}$$

where *n* is the number of elements in which the sample of ${H}_{s}$ is discretized; $\Delta {H}_{s}$ is the width of each element.

In this study a non-parametric empirical PDF from a ${\mathrm{H}}_{\mathrm{s}}$ sample over the ${q}_{99}$ for both data sets is calculated using the Kernel density estimation. Bias correction allows to improve the agreement between the GWP_GCM_ and the reference data, reducing the variability between members (Supplementary Fig. 2).

### Robustness of the projected change

The uncertainty assessment of the projected changes is based on a method proposed in the AR5 report^72^. In particular, the robustness of the change is addressed considering, first, its significance and, second, the agreement in the sign of change between the members of the ensemble. Grid nodes (Supplementary Fig. 1) in which the change is statistically significant at 95% confidence level (α = 0.05; Eq. 4) in more than 50% of the ensemble members and at least 80% of the them agree in the sign of change are considered as robust and are stippled. If both conditions are not met, grid nodes are not stippled. Cases in which the first condition is met but not the second one are shown without stippling (instead of being masked as white^72^) to facilitate the spatial comprehension of the results.

4 $$\left(1-\alpha \right) {confidence \,intervals:} \stackrel{-}{X}\pm\, {t}_{\frac{\alpha }{2},n-1}\cdot \frac{s}{\sqrt{n}}$$

where $\alpha$ is the significance level, $n$ is the sample size and $s$ is the standard deviation normalized by $n-1$.

### Statistical extreme model

This study is carried out following the Annual Maxima Method (AMM)^30^, a block maxima approach in which AM from hourly time series are fit to a generalized extreme value distribution (GEVD; Eq. 5).

5 $$F(x)=exp\left\{-{\left[1+\xi \left(\frac{x-\mu }{\sigma }\right)\right]}^{-1/\xi}\right\}$$

where $\mu$ is the location parameter, $\sigma$ is the scale parameter and $\xi$ is the shape parameter. The sign of $\xi$ provides information about the behaviour of the tail of the distribution, i.e. $\xi$ < 0 indicates a bounded upper limit tail (Weibull family), $\xi$ > 0 denotes a heavy tail (Fréchet family) and $\xi$ = 0 a light tailed case (Gumbel family).

In particular, we apply the simplified extreme value distribution family of the GEVD: type I EVD (commonly known as Gumbel distribution; Eq. 6).

6 $$F(x)=exp\left[-exp\left(-\frac{x-\mu }{\sigma }\right)\right]$$

### Goodness of fit of the extreme model

The goodness-of-fit of the Gumbel distribution is assessed with the Anderson–Darling test^49^ (AD). This test statistic (Eq. 7)^73^ measures the distance between the proposed distribution with the estimated parameters and the empirical distribution, introducing as well a weight function that gives heavier weights in both tails to improve the performance. AD test is applied to evaluate whether the null hypothesis (*H*_*0*_) is rejected or not at the 95% confidence level (α = 0.05). *H*_*0*_ states that the data follows a Gumbel distribution and *H*_*1*_, the alternative hypothesis, the contrary. The null hypothesis is rejected or not depending on the comparison between the p value and the considered significance level.

7 $${A}_{n}^{2}=-n-\sum_{i=1}^{n}\frac{2i-1}{n}\left[\mathrm{log}\left(F\left({X}_{i}\right)\right)+\mathrm{log}\left(1-F\left({X}_{n+1-i}\right)\right)\right]$$

where $\left\{{X}_{1}<\dots <{X}_{n}\right\}$ are the ordered sample data points and $n$ is the size of the sample.

### Validation with buoy data

The significant wave height return values are validated against buoy data. All the selected buoys meet a number of quality requirements to ensure a proper performance of the EVA and an adequate comparison with the outcomes of the numerical projections, resulting in a final set of fifty-two buoys. These requirements are listed below:

- The buoy should be moored at a water depth higher than 50 m to ensure that waves do not propagate in shallow waters and avoid non-linear processes.
- The buoy should be moored at a distance to the coast higher than 20 km to take into account the spatial resolution of the global wave climate simulations (i.e. one-degree). Nevertheless, the limited number of buoys in the SH makes us to relax this requirement to ensure a validation of southern extreme waves, setting the distance limit at 5 km for buoys located in the SH.
- Annual maxima are selected differently depending on the latitude of the buoy. For buoys located above 40°N, AM are selected from years presenting more than a 60% of the hourly data in the boreal winter months (i.e. January, February and December). For buoys located below 40°S, AM are selected in years presenting more than a 60% of the hourly data in the austral winter months (i.e. June, July and August). For the rest of buoys, AM are selected in years presenting more than 60% of the hourly data.
- The resulting annual maxima sample should have at least twenty values to carry out the EVA.

## Supplementary information

Supplementary Information.
