# Supplementary material for: Future behavior of wind wave extremes due to climate change
Source: Sci Rep. 2021 Apr 12;11:7869. doi: 10.1038/s41598-021-86524-4 (PMC8042069; doi:10.1038/s41598-021-86524-4)
Supplement: Supplementary file 1 — Supplementary Information. [file 41598_2021_86524_MOESM1_ESM.pdf]

# Future behavior of wind wave extremes due to climate change

Hector Lobeto[1], Melisa Menendez[1*], Iñigo J. Losada[1]

[1]*IHCantabria - Instituto de Hidráulica Ambiental de la Universidad de Cantabria*

**Correspondence to menendezm@unican.es*

## Supplementary material

### Atmosphere-Ocean General climate model data

This work makes use of a CMIP5-based multi-model ensemble consistent of seven atmosphere-ocean general circulation models (GCMs) (Supplementary Table 1). The criterion to select the models is defined by the need of producing hourly time series of $H_s$ to better capture the extremes. Therefore, we look for GCMs that provide surface winds and sea ice coverage fields with the maximum time resolution as possible. All the GCMs provide 3-hourly winds and daily ice coverage fields (except from HadGEM2-ES that provides monthly ice) for the historical and projected periods, both for RCP4.5 and RCP 8.5 greenhouse gas emission scenarios[1]. A single run of each GCM is simulated to reduce the storage capacity demanded by the hourly $H_s$ series needed, which is sustained by the low variability between wave projections from runs of the same model[2]. In particular, the r1i1p1 member of each realization ensemble is selected.

In addition to the spatial resolution, the independency of the models is analyzed to ensure the reliability of the ensemble mean results. This issue is addressed on the basis of the classification of GCMs proposed by Knutti et al., in 2013[3] in terms of the degree of dissimilarity between pairs of individual models with regard to a multivariate description of the natural variability[4]. The similarity between models is defined using the Kullback-Leibler divergence distance metric considering the seasonal cycle, interannual variations, annual mean climatology and spatial correlation from temperature and precipitation monthly fields, both for the control state period and for the projected changes under RCP8.5 scenario by the end of the century. A hierarchical clustering is applied to define the relationship between members, presenting the results as a dendrogram. HadGEM2-ES and ACCESS1.0 are the closest in the control state, which can be related to sharing the same atmospheric model. Concerning the projected changes, there are no strong enough similarities that may cause a bias in the results, not even for the two models found to be similar in the control period.

| ***GCM*** | ***Institution*** | ***Country*** | ***Atmospheric resolution (lat x lon)*** |
|---|---|---|---|
| ***MIROC5*** | MIROC | Japan | 1,40º x 1,40º |
| ***IPSL-CM5A-MR*** | Institut Pierre-Simon Laplace | France | 1,25º x 1,25º |
| ***GFDL-ESM2G*** | NOAA Geophysical Fluid Dynamics Laboratory | USA | 2,00º x 2,50º |
| ***CNRM-CM5*** | Centre National de Recherches Météorologiques | France | 1,40º x 1,40º |
| ***CMCC-CM*** | Centro Euro-Mediterraneo per I Cambiamenti Climatici | Italy | 0,75º x 0,75º |
| ***ACCESS1.0*** | CSIRO-BOM | Australia | 1,25º x 1,90º |
| ***HadGEM2-ES*** | Met Office Hadley Centre | UK | 1,25º x 1,90º |

***Supplementary Table 1*** *Main characteristics of the selected Atmosphere-Ocean General Circulation Models.*

## Dynamical wave projections

A series of global wave projections ($GWP_{GCM}$) are dynamically simulated considering sea surface wind fields and ice coverage outputs from the ensemble members as inputs of the wave generation model. The third-generation wave model WaveWatchIII (WW3) version 4.18[5] is used to run the simulations. The main features of the numerical scheme used in this work are summarized below:

- Parametrization TEST451[6].
- Continuous ice concentration blocking from 0.25 (no blocking) to 0.75 (total blocking).
- Discrete Interaction Approximation[7] (DIA)
- Depth-inducing breaking following Battjes and Janssen approach[8].
- SHOWEX bottom friction formulation[9].
- Coastal reflexion equal to 0.05.
- Energy flux reduction due to islands or any other coastal obstacles smaller than cell size[10].
- Third-order Ultimate Quickest propagation scheme[11].

Significant wave height hourly time series are obtained globally at 1° spatial resolution. Grid nodes with less than 60% of the hourly data due to the presence of ice for any of the $GWP_{GCM}$ are not selected. Supplementary Figure 1 shows the final 31591 selected grid nodes.

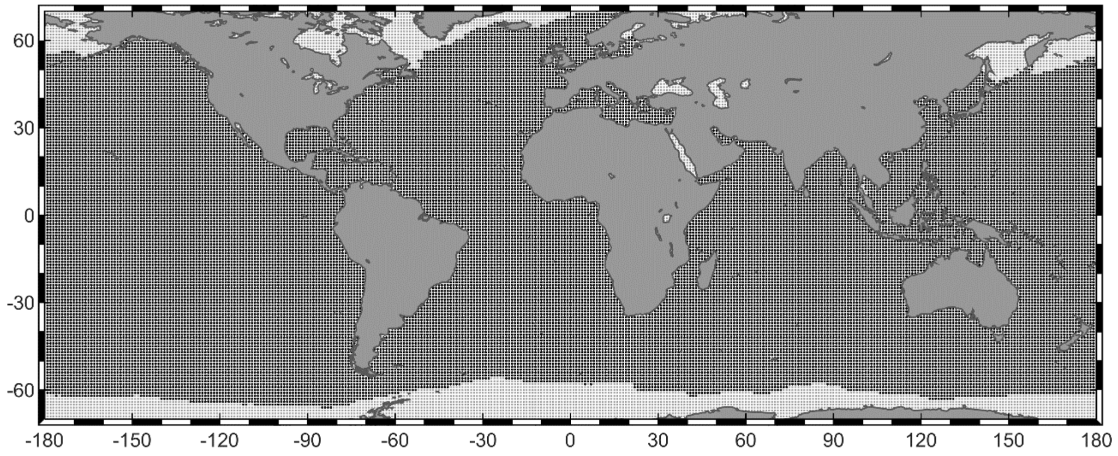


***Supplementary Figure 1*** *Black dots represent the selected grid nodes to study the projected changes in wave height extremes. Figure generated with MATLAB R2020a ([https://es.mathworks.com/products/matlab.html](https://es.mathworks.com/products/matlab.html)).*

## Bias correction (BC)

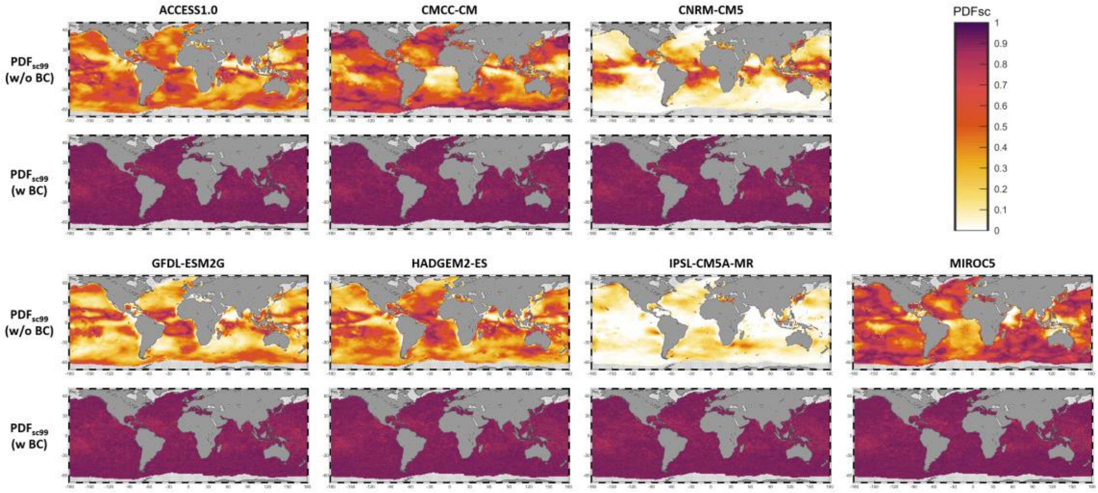


***Supplementary Figure 2*** *Agreement between the distribution of $H_s$ above the $q_{99}$ before applying the bias correction (w BC panels) and after correcting the bias (w/o BC panels) under RCP8.5 scenario. Figure generated with MATLAB R2020a ([https://es.mathworks.com/products/matlab.html](https://es.mathworks.com/products/matlab.html)).*

## Extreme value analysis

*GEV parameters assessment*

Global GEV parameters and their projected variation by the end of the century under RCP8.5 scenario are shown in Supplementary Figure 3. The location parameter (μ), an indicator of the central values of the extreme distribution, increases with latitude until reaching its highest values in the extratropical region and, in particular, in the North Atlantic Ocean, where μ reaches values close to 14m (Supplementary Figure 3a). There is a robust agreement concerning the future decrease in μ in the Northern Hemisphere, showing local decreases close to 1m in the North Atlantic Ocean and around the Japanese Archipelago. The northernmost Pacific Ocean and the northwestern Atlantic are the most remarkable exceptions found within this decreasing pattern (Supplementary Figure 3d). A robust projected change is also obtained in the Southern Ocean although with the opposite sign (i.e. increase in μ).

The spatial distribution of the scale parameter (σ) is quite similar to the location's (i.e. higher values at higher latitudes) although showing some relevant differences such as the very high σ values found around Japan (Supplementary Figure 3b). Concerning future changes (Supplementary Figure 3e), a higher noise in the sign of change with respect to μ can be observed, making it difficult to find other consistent general conclusions beyond an increase in the Southern Ocean. In spite of this, some relevant aspects such as the robust decrease found in the tropical southeastern Indian Ocean and the loss of a clear decreasing change pattern in the North Atlantic Ocean can be pointed out.

The shape parameter (ξ) presents positive values (i.e. heavy-tailed distribution) in tropical cyclone (TC) activity regions and TC-induced swell affecting zones (Caribbean Sea, West Pacific, South-West Indian Ocean; Supplementary Figure 3c). Negative values (i.e. bounded upper tail distribution) can be observed throughout the global ocean, being the northeastern Atlantic, southeastern Pacific and south Indian the regions which present the clearest negative patterns.

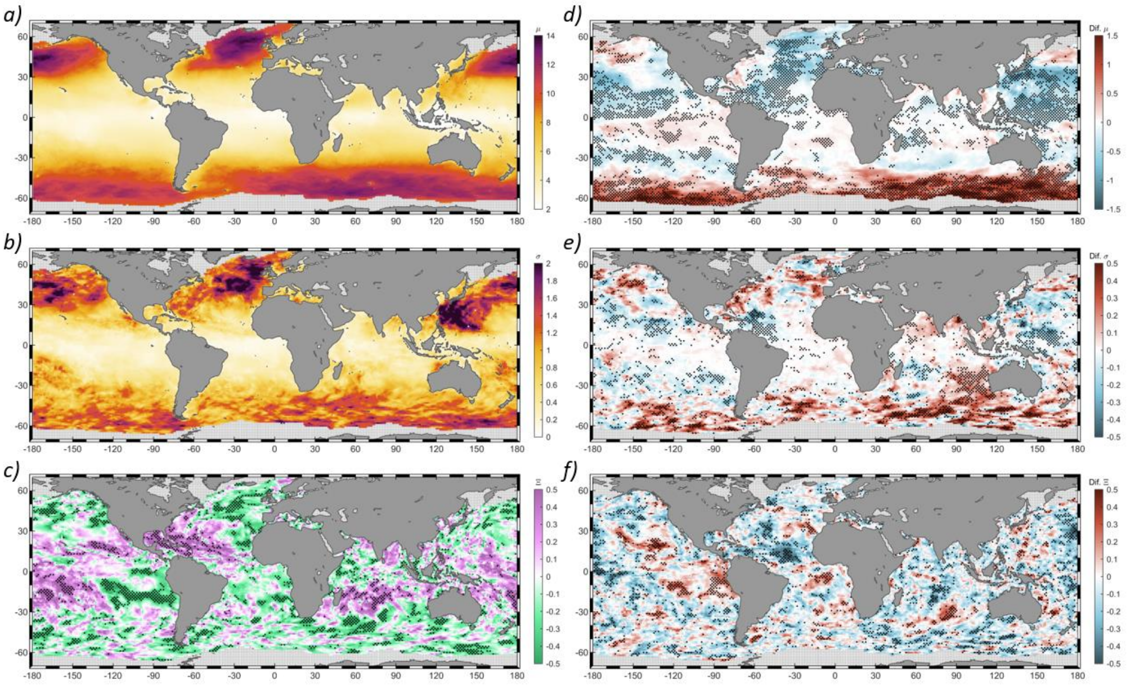


***Supplementary Figure 3*** *Location (a), scale (b) and shape (c) GEV parameters from GOW2 hindcast. Multi-model ensemble mean change in the location (d), scale (e) and shape (f) parameters by the end of the century (2081-2100 relative to 1986-2005). Stippling in (c) denotes significant fit of the shape parameter at 95% confidence level. Stippling in right panels denotes changes statistically significant at 95% confidence level in at least 50% of the members and an agreement in the sign of change in more than 80% of the members. Figure generated with MATLAB R2020a (https://es.mathworks.com/products/matlab.html).*

*Goodness-of-fit of the extreme model*

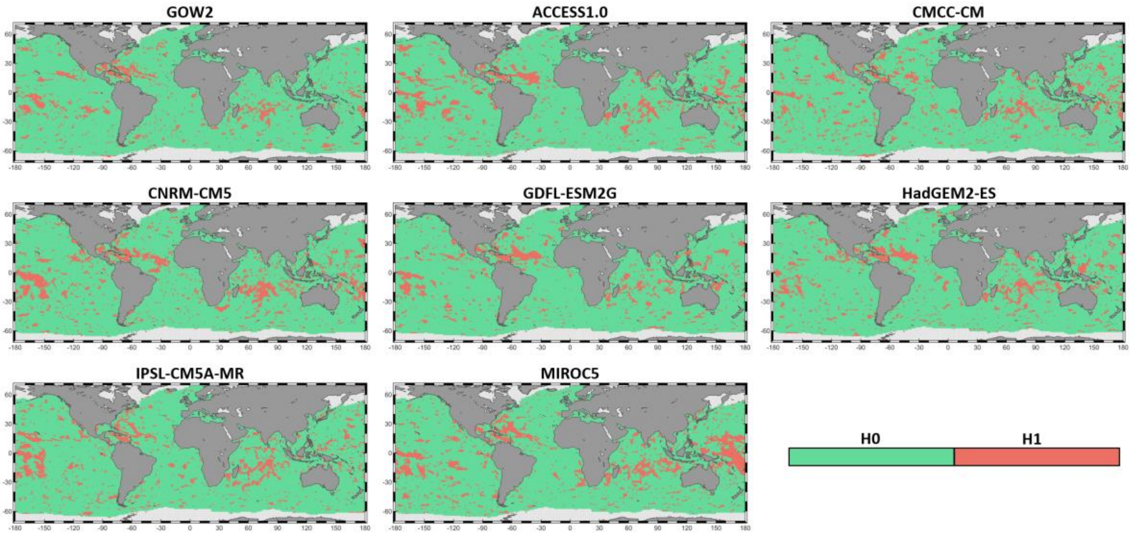


***Supplementary Figure 4*** *Anderson-Darling test statistic at 95% confidence level (α=0.05) for GOW2 and every member of the ensemble under RCP8.5 scenario. Orange grid points indicate where the null hypothesis ($H_0$) is rejected at 95% confidence level (α=0.05) for each GCM. Figure generated with MATLAB R2020a (https://es.mathworks.com/products/matlab.html).*

| MIROC5 | IPSL-CM5A-MR | GFDL-ESM2G | CNRM-CM5 | CMCC-CM | ACCESS1 | HadGEM2-ES |
|---|---|---|---|---|---|---|
| 14.7 | 11.8 | 10.4 | 13.3 | 11.8 | 12.1 | 11.6 |

***Supplementary Table 2*** *Proportion of the global ocean (%) where the null hypothesis ($H_0$) is rejected at 95% confidence level (α=0.05) for each GCM.*

*Tropical cyclone representation*

The relation between the maximum and the median $H_s$ before the bias correction is calculated globally. The TC simulation performance is assessed comparing the results against the GOW2 wave hindcast, which has already been validated for extreme TC-induced waves[12].

Results show that all members are able to simulate the extreme wave climate induced by TC activity (dark red colors from Supplementary Figure 5). However, there is a general underestimation of the magnitude of extremes, existing notable discrepancies between members. For example, MIROC5 is the model that best represent the TCs, showing extreme waves in all the regions affected by TCs. CNRM-CM5 and CMCC-CM also show extreme waves in all these regions, although with a clear underestimation with respect to MIROC5. The rest of the models show a great underestimation of TC-induced extreme wave climate, especially in the westernmost Pacific, the world's region most affected by this kind of events.

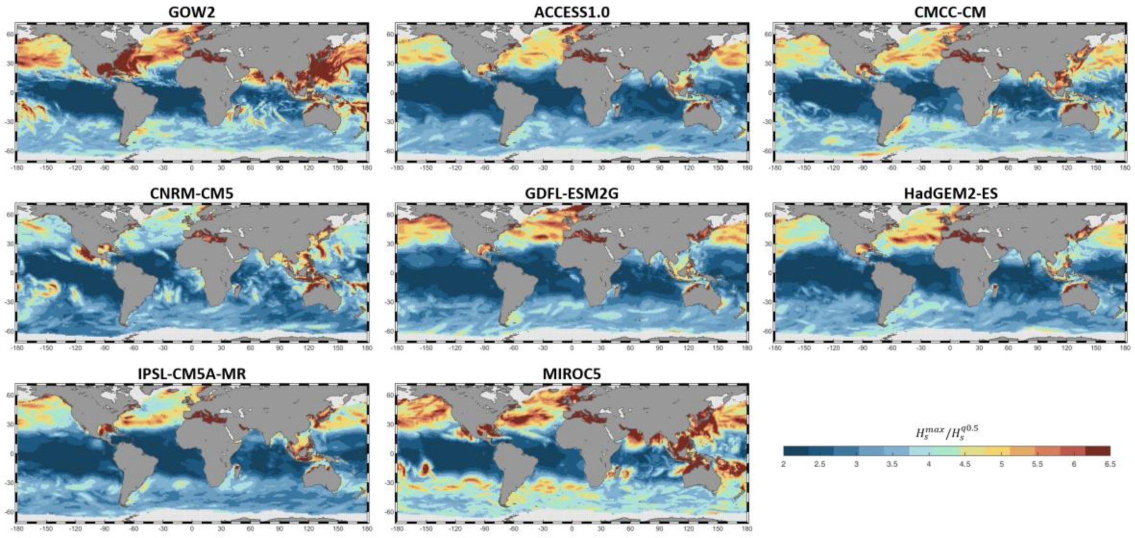


***Supplementary Figure 5*** *Relation between the maximum* $H_s$ *(*$H_s^{max}$*) and the median* $H_s$ *(*$H_s^{q0.5}$*) before applying the bias correction for each GCM and for the reference wave hindcast. Figure generated with MATLAB R2020a (https://es.mathworks.com/products/matlab.html).*

## Regionalization of the global ocean

The global ocean is discretized in ocean regions to better understand the projected changes. Although a division with straight lines is far from the physical reality as ocean basins are not independent units, the assessment of the average changes and their robustness not only at global scale, but also in each of the regions included in the analysis, helps to provide a more detailed and structured description of the projected wave climate changes. The division is based on the regionalization proposed by Alves in 2006[13] attending to the swell generation criteria, which have been extensively cited in wave climate studies[14–16]. Thus, at first, each ocean basin (i.e. Pacific, Atlantic and Indian) is divided in four regions: extra-tropical north (above 30ºN), tropical north (30ºN to equator), tropical south (equator to 30ºS) and extra-tropical south (below 30ºS). Then, this initial division is modified to better fit with the spatial change pattern found after comparing present and future extreme wave height and to give more accurate aggregate results. First, the latitudinal threshold that separates tropical and extra-tropical regions is risen from 30º to 35º, defining the extra-tropical north Pacific (ETNP) and the extra-tropical north Atlantic (ETNA) regions. Then, all extra-tropical south regions are joint together to constitute the Southern Ocean (SO). The eastern transition between the tropical north Atlantic (TNA) and the tropical south Atlantic (TSA) is also slightly modified. Concerning the Pacific basin, a new wedge-shaped region is introduced in the tropical eastern Pacific (TEP) between tropical north (TNP) and tropical south (TSP) regions. Finally, while tropical north Indian (TNI) remains unaltered, the tropical south Indian Ocean is divided in two regions: tropical southeastern Indian (TSEI) and tropical southwestern Indian (TSWI).

## Validation of significant wave height return values

A set of fifty-two buoy records are selected to conduct the validation (Supplementary Table 3). Supplementary Figure 6 and Supplementary Table 4 summarize the results before and after applying the bias correction. A mean relative error (MRE) lower than 20% is obtained after bias correction for all the return periods. The analysis for the non-bias-corrected $H_s$ return values evidences a clear reduction of the error after the correction.

| Name | Lon (°) | Lat (°) | Depth (m) | Name | Lon (°) | Lat (°) | Depth (m) |
|---|---|---|---|---|---|---|---|
| NDBC-41001 | -72.63 | 34.60 | 4462 | NDBC-46041 | -124.74 | 47.35 | 131 |
| NDBC-41002 | -75.16 | 32.07 | 4297 | NDBC-46050 | -124.51 | 44.64 | 127 |
| NDBC-41010 | -78.55 | 28.90 | 887 | NDBC-46132 | -127.93 | 49.74 | 2047 |
| NDBC-42001 | -89.66 | 25.86 | 3217 | NDBC-46184 | -138.86 | 53.91 | 3380 |
| NDBC-42002 | -94.04 | 25.63 | 3125 | NDBC-46042 | -122.44 | 36.77 | 1890 |
| NDBC-42003 | -85.65 | 25.98 | 3292 | NDBC-46185 | -129.79 | 52.42 | 220 |
| NDBC-42019 | -95.35 | 27.91 | 83 | NDBC-46204 | -128.76 | 51.38 | 223 |
| NDBC-42020 | -96.70 | 26.96 | 83 | NDBC-46206 | -126.00 | 48.83 | 119 |
| NDBC-42036 | -84.51 | 28.51 | 52 | NDBC-46207 | -129.91 | 50.87 | 2088 |
| NDBC-42039 | -86.03 | 28.80 | 291 | NDBC-46208 | -132.70 | 52.51 | 2922 |
| NDBC-42040 | -88.22 | 29.21 | 190 | NDBC-51001 | -162.14 | 23.94 | 4869 |
| NDBC-44004 | -70.50 | 38.48 | 3182 | NDBC-51002 | -157.75 | 17.11 | 5029 |
| NDBC-44005 | -69.15 | 43.19 | 205 | NDBC-51003 | -160.65 | 19.18 | 4920 |
| NDBC-44008 | -69.34 | 40.50 | 63 | NDBC-51004 | -152.40 | 17.52 | 5082 |
| NDBC-44011 | -66.60 | 41.11 | 86 | QLD-Brisbane | 153.63 | -27.49 | 77 |
| NDBC-46001 | -148.02 | 56.30 | 4124 | Met Office-K1 | -12.40 | 48.70 | 1871 |
| NDBC-46002 | -130.47 | 42.59 | 3438 | Met Office-K2 | -13.30 | 51.00 | 1994 |
| NDBC-46005 | -131.01 | 46.07 | 2721 | Met Office-Brittany | -8.50 | 47.50 | 2108 |
| NDBC-46006 | -137.44 | 40.79 | 4294 | Met Office-K5 | -11.40 | 59.10 | 2011 |
| NDBC-46012 | -122.88 | 37.36 | 206 | PdE-Silleiro | -9.40 | 42.12 | 301 |
| NDBC-46013 | -123.31 | 38.23 | 125 | PdE-Cadiz | -6.63 | 36.66 | 450 |
| NDBC-46022 | -124.55 | 40.74 | 512 | MHL-Byron Bay | 153.72 | -28.77 | 65 |
| NDBC-46028 | -121.87 | 35.73 | 1075 | MHL-Coffs Harbour | 153.27 | -30.35 | 69 |
| NDBC-46029 | -124.51 | 46.12 | 137 | MHL-Crowdy Head | 152.86 | -31.82 | 64 |
| NDBC-46035 | -177.67 | 57.06 | 3682 | MHL-Port Kembla | 151.02 | -34.47 | 82 |
| NDBC-46036 | -133.93 | 48.35 | 3548 | MHL-Sydney | 151.42 | -33.78 | 97 |

**Supplementary Table 3** Main characteristics of the selected buoys: name, longitude, latitude and depth

| | | $H_s^5$ | $H_s^{20}$ | $H_s^{50}$ |
|---|---|---|---|---|
| w BC | Mean relative error (%) | 13.1 | 17.5 | 19.6 |
| | Interquartile range of relative error (%) | 6.9-17.9 | 9.6-24.0 | 11.5-26.9 |
| | Mean square error (m) | 2.3 | 7.0 | 11.9 |
| w/o BC | Mean relative error (%) | 28.2 | 33.2 | 35.4 |
| | Interquartile range of relative error (%) | 18.9-34.9 | 24.2-41.5 | 27.3-44.5 |
| | Mean square error (m) | 8.0 | 18.7 | 28.7 |

***Supplementary Table 4*** *Relative error (%), interquartile range of relative error and mean-square error in significant wave height return values calculated from the dynamical projections with (w) and without (w/o) BC with respect to return values from buoy records.*

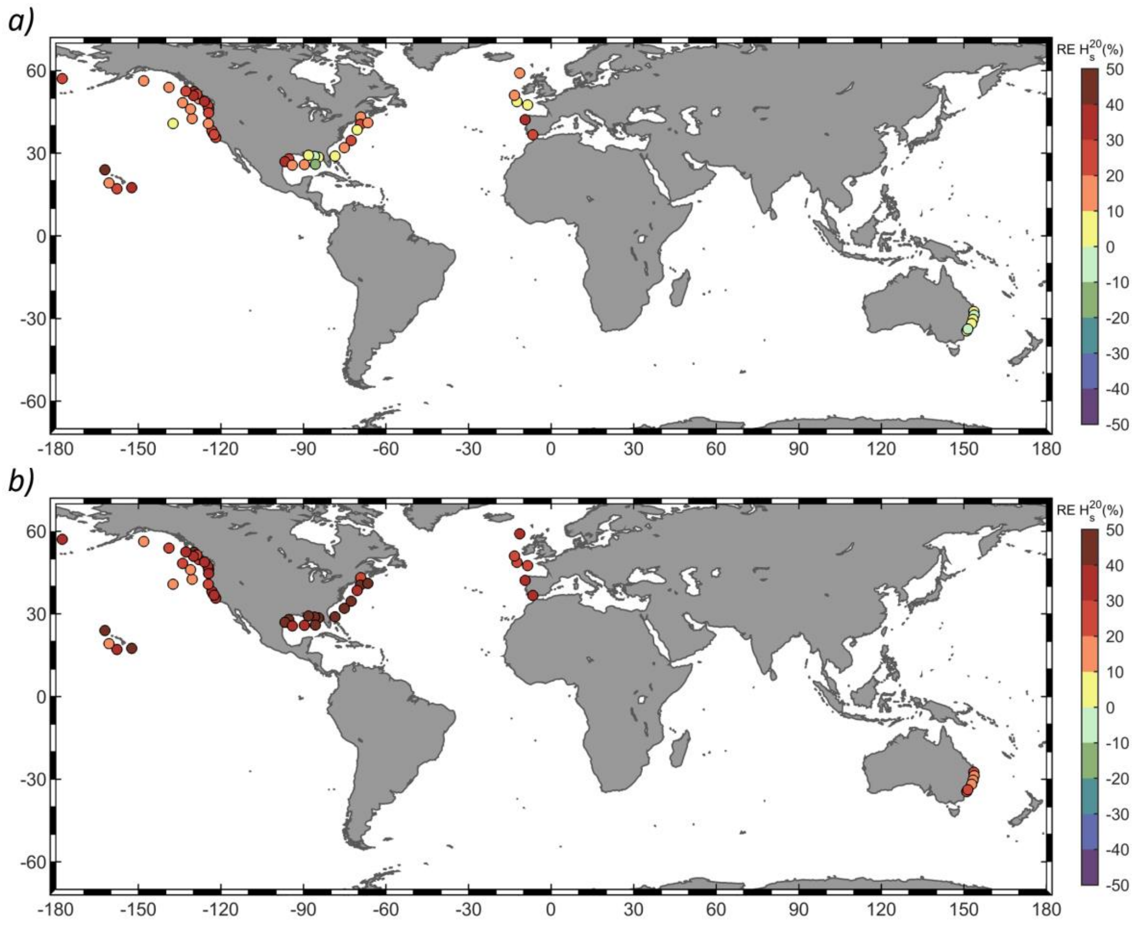


***Supplementary Figure 6*** *Relative error (%) in significant wave height 20-year return values calculated from the dynamical projections with (a) and without (b) BC with respect to return values from buoy records. Figure generated with MATLAB R2020a (https://es.mathworks.com/products/matlab.html).*

## Extreme wave climate future changes under RCP8.5 scenario

In addition to the changes in the 20-year return period $H_s$ extremes (Figure 2c; Table 1), future changes in 5-, 50- and 100-year return period events under RCP8.5 scenario are also studied (Supplementary Figure 7d-f; Table 1). The main conclusions derived from the found spatial change pattern agree with those already explained for $H_s^{20}$. Concerning the magnitude of the projected changes, the Southern Ocean presents an expected future increase of +0.6m, +0.9m and +0.9m for $H_s^{5}$, $H_s^{50}$ and $H_s^{100}$, respectively. The Atlantic Ocean exhibit a decreasing pattern in the Northern Hemisphere, especially robust in the tropical region for all return periods (above 28% of the region show robust changes), with projected decreases of -0.3m, -0.4m and -0.4m. On the other hand, the high uncertainty in TSA (10% of robust change for all return periods) precludes the possibility of reaching any strong conclusion. Along the tropical Pacific, a consistent decreasing change pattern can be observed with the only exception of TEP region, where a highly uncertain increase is found (less than 10% of robust change). TNP shows an average projected change of -0.5m, -0.6m and -0.6m for $H_s^{5}$, $H_s^{50}$ and $H_s^{100}$, and TSP presents an average change of -0.2m, -0.3m and -0.4m for and $H_s^{5}$, $H_s^{50}$ and $H_s^{100}$, respectively. The Indian Ocean is characterized by heterogeneity in the sign of change. Thus, while in TSWI an average decrease of -0.1m for $H_s^{5}$, -0.2m for $H_s^{50}$ and -0.2m for $H_s^{100}$is obtained, TSEI shows the opposite sign of change with +0.1m, +0.3m and +0.4m, respectively. Again, the high uncertainty found in TNI makes it very difficult to assert a robust conclusion. In addition, the projected change in the return period associated to present-day extreme wave heights by the end of the century is also shown (Supplementary Figure 8).

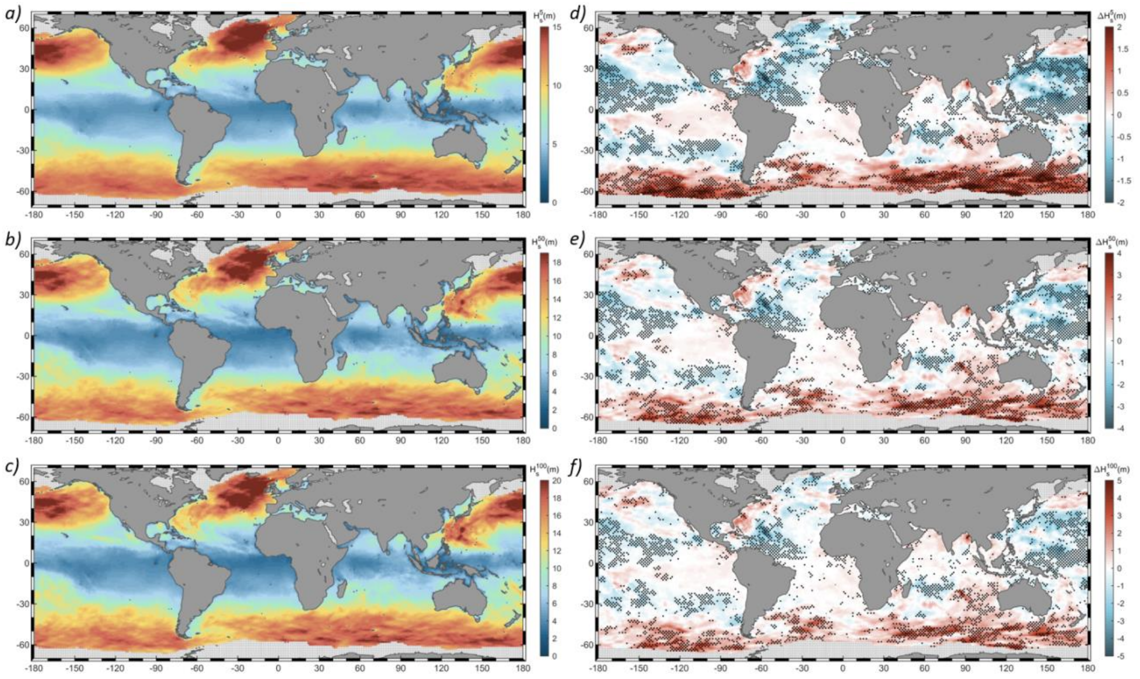

***Supplementary Figure 7*** *$H_s^5$ (a), $H_s^{50}$ (b) and $H_s^{100}$ (c) from GOW2 hindcast. Multi-model ensemble mean change in $H_s^5$ (d), $H_s^{50}$ (e) and $H_s^{100}$ (f) (in meters) under RCP8.5 scenario. Stippling denotes changes statistically significant at 95% confidence level in at least 50% of the members and an agreement in the sign of change in more than 80% of the members. Figure generated with MATLAB R2020a (https://es.mathworks.com/products/matlab.html).*

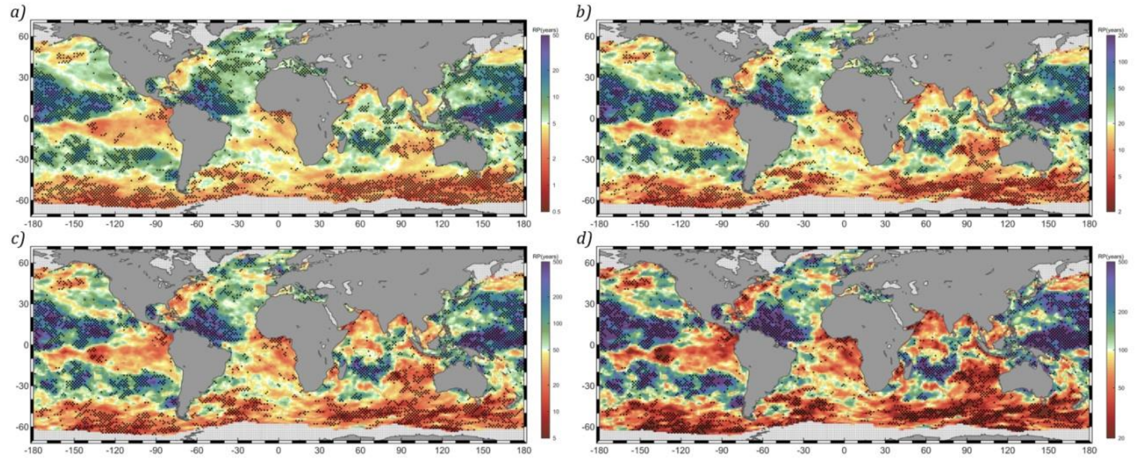

***Supplementary Figure 8*** *Projected return period by 2100 of present-day $H_s^5$ (a), $H_s^{20}$ (b), $H_s^{50}$(c) and $H_s^{100}$ (d). Stippling indicates the same as in Sup. Figure 7. Figure generated with MATLAB R2020a (https://es.mathworks.com/products/matlab.html).*

## Extreme wave climate future changes under RCP4.5 scenario

Future changes under RCP4.5 scenario have a very similar special pattern for all the analyzed return periods (Supplementary Figure 9; Table 1). Nevertheless, the uncertainty increases for wave extremes with a lower probability of occurrence so that the proportion of the global ocean that shows robust changes reduces from 22% for $H_s^5$ to 16% for $H_s^{100}$. Results for $H_s^{20}$ (Supplementary Figure 9b; Table 1) indicate a robust expected decrease in the North Atlantic Ocean excluding the westernmost part of the basin, where a consistent positive change is found. The proportion of the region with a robust decrease is 13% in ETNA and 22% in TNA, with averaged projected changes of -0.2m and -0.0m, respectively. TSA shows a positive change characterized by a 16% of robust changes and an average increase of +0.1m. Concerning the Pacific Ocean, ETNP presents a low-robust increase (12%) with an average change of +0.1m. The tropical region shows a heterogenous spatial pattern so that

while a decrease is expected in TNP and TSP (average of -0.2m in both regions), a very low-robust positive projected change is expected in TEP (8%). The change pattern in the Indian Ocean is characterized by a high uncertainty in TNI (11%) and heterogeneity in the sign of change in the tropical south region. Thus, while a robust increase can be observed in TSEI (19% and average change of +0.2m), TSWI presents areas with robust projected positive (e.g. Mozambique Channel) and negative (e.g. east of Madagascar) changes, resulting in an average projected change of -0.0m for all return periods. Finally, a consistent increase in the Southern Ocean characterized by a 20% of robust changes and an average of +0.4m is obtained.

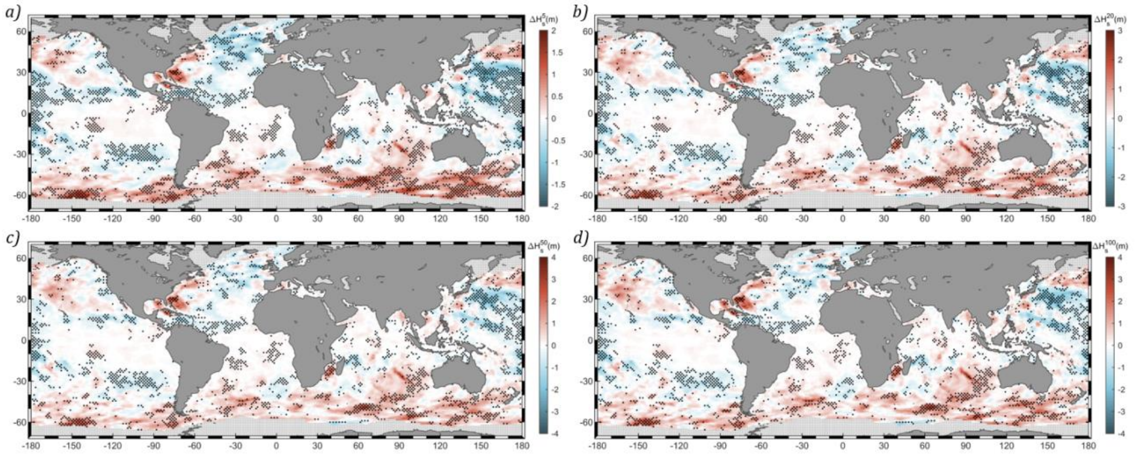


***Supplementary Figure 9*** *Multi-model ensemble mean change in $H_s^5$ (a), $H_s^{20}$ (b), $H_s^{50}$ (c) and $H_s^{100}$ (in meters) under RCP4.5 scenario. Stippling denotes changes statistically significant at 95% confidence level in at least 50% of the members and an agreement in the sign of change in more than 80% of the members. Figure generated with MATLAB R2020a ([https://es.mathworks.com/products/matlab.html](https://es.mathworks.com/products/matlab.html)).*

In this work we also carry out a comparison between the spatial pattern obtained for projected changes in mean and extreme wave height by the end of the century under RCP4.5 (Supplementary Figure 10) scenario. Despite results exhibit a concordance in the sign of change between both conditions in approximately two thirds of the global ocean, a general great uncertainty can also be observed (only 9% of the area show robust change for mean and extreme wave climate). In this regard, while the Southern Ocean (increase) and the north tropical western Pacific (decrease) are the regions which present the most robust agreement, the extra-tropical north Pacific and tropical southeastern Indian are the areas with the most evident disagreement. Similarly, the North Atlantic Ocean shows a concordance in the sign of change in most of the basin that breaks down due to the positive projected changes obtained for extremes in the westernmost part of the region.

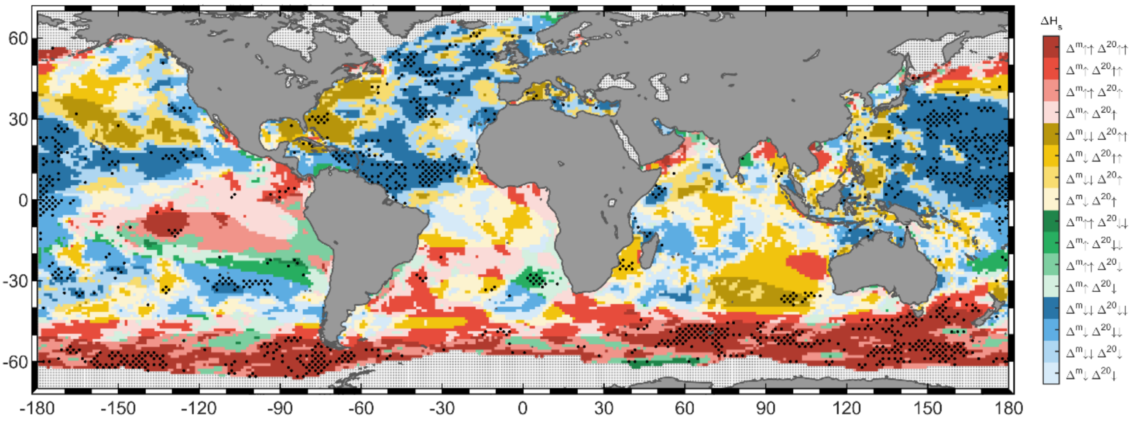


***Supplementary Figure 10*** *Comparison of projected changes in mean ($\Delta^m$) and extreme ($\Delta^{20}$) significant wave height under RCP4.5 scenario by the end of the century (2081-2100 relative to 1986-2005). Colors represent the combinations of sign of change between mean and extreme wave conditions: red indicates an increase in both conditions, blue represent a decrease in both conditions, yellow represent a decrease in mean and an increase in extreme conditions and green represents an increase in mean and a decrease in extreme conditions. For each combination, one arrow indicates a relative change lower than the global median and two arrows indicate relative changes higher than the global median. The global median is obtained as the median of the*

*relative changes with the same sign as the analyzed variation. Stippling denotes changes statistically significant at 95% confidence level in at least 50% of the members and an agreement in the sign of change in more than 80% of the members for both mean and extreme significant wave height. Figure generated with MATLAB R2020a ([https://es.mathworks.com/products/matlab.html](https://es.mathworks.com/products/matlab.html)).*

## Mean wave climate future changes

Projected changes in annual mean significant wave height ($H_s^m$) are calculated as the difference between future and present-day $H_s^m$. The uncertainty assessment follows the same method as for projected changes in extremes (see Methods).

Changes in $H_s^m$ under RCP8.5 scenario (Supplementary Figure 11a) exhibit a general decreasing pattern in the Northern Hemisphere. In this regard and in line with previous studies, the North Atlantic and the northwestern Pacific show a robust projected change[14,17,18]. The northeastern Pacific presents as well a consistent decrease although with a higher uncertainty compared to the previous regions. Concerning the Southern Hemisphere, a heterogenous spatial change pattern can be observed. The Southern Ocean and the tropical eastern Pacific present a robust increase, which agrees with the projected changes found in previous studies[14,17,18]. Contrarily, a robust negative change can also be observed in the Indian Ocean. Results for the RCP4.5 (Supplementary Figure 11b) show a pretty similar spatial change pattern with respect to the RCP8.5 scenario although with a notable increase in the uncertainty (from 63% to 46% of the global ocean showing robust changes). Despite the north Atlantic, northwestern Pacific, Southern Ocean and tropical eastern Pacific maintain a robust agreement in the projected changes, there is a remarkable increase in the uncertainty in regions such as the Indian Ocean.

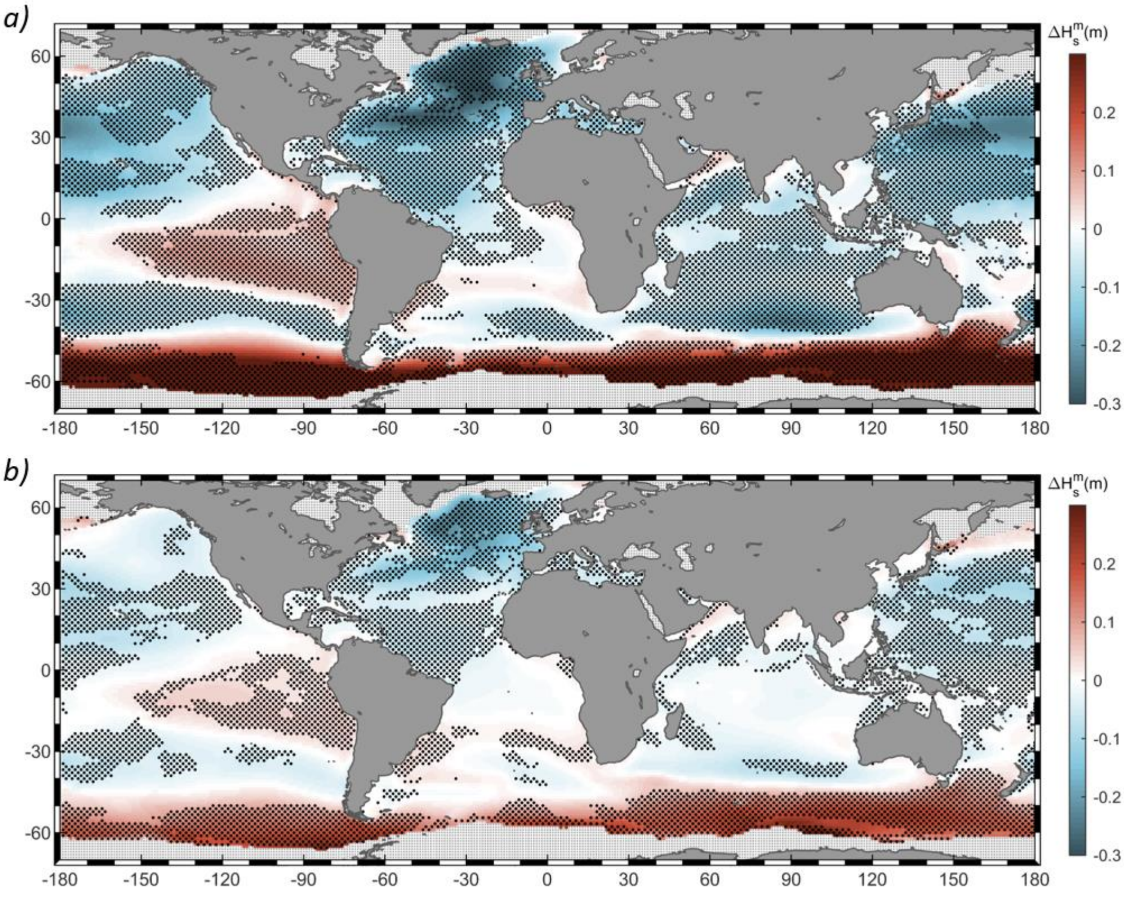


***Supplementary Figure 11*** *a) Multi-model ensemble mean change in $H_s^m$ under RCP8.5 scenario. b) Multi-model ensemble mean change in $H_s^m$ under RCP4.5 scenario. Stippling denotes changes statistically significant at 95% confidence level in at least 50% of the members and an agreement in the sign of change in more than 80% of the members. Figure generated with MATLAB R2020a ([https://es.mathworks.com/products/matlab.html](https://es.mathworks.com/products/matlab.html)).*

## References


1. Cubasch, U. *et al.* Introduction. in *Climate Change 2013: The Physical Science Basis. Contribution of Working Group I to the Fifth Assessment Report of the Intergovenermnetal Panel on Climate Change* (eds. Stocker, T. F. et al.) (2013). doi:10.2753/JES1097-203X330403

2. Semedo, A. *et al.* CMIP5-derived single-forcing, single-model, and single-scenario wind-wave climate ensemble: Configuration and performance evaluation. *J. Mar. Sci. Eng.* **6**, (2018).

3. Knutti, R., Masson, D. & Gettelman, A. Climate model genealogy: Generation CMIP5 and how we got there. *Geophys. Res. Lett.* **40**, 1194–1199 (2013).

4. Masson, D. & Knutti, R. Climate model genealogy. *Geophys. Res. Lett.* **38**, 1–4 (2011).

5. Tolman, H. L. User manual and system documentation of WAVEWATCH III. **3**, 98–106 (2014).

6. Ardhuin, F. *et al.* Semiempirical dissipation source functions for ocean waves. Part I: Definition, calibration, and validation. *J. Phys. Oceanogr.* (2010). doi:10.1175/2010JPO4324.1

7. Hasselmann, S., Hasselmann, K., Allender, J. H. & Barnett, T. P. Computations and parameterizations of the nonlinear energy transfer in a gravity-wave spectrum. Part II: parameterizations of the nonlinear energy transfer for application in wave models. *J. PHYS. Ocean.* (1985). doi:10.1175/1520-0485(1985)015<1378:capotn>2.0.co;2

8. Battjes, J. A. & Janssen, J. P. F. M. Energy loss and set-up due to breaking of random waves. in *Proceedings of the Coastal Engineering Conference* (1979). doi:10.9753/icce.v16.32

9. Ardhuin, F., O'Reilly, W. C., Herbers, T. H. C. & Jessen, P. F. Swell transformation across the continental shelf. Part I: Attenuation and directional broadening. *J. Phys. Oceanogr.* (2003). doi:10.1175/1520-0485(2003)033<1921:STATCS>2.0.CO;2

10. Tolman, H. L. Treatment of unresolved islands and ice in wind wave models. *Ocean Model.* **5**, 219–231 (2003).

11. Leonard, B. P. A stable and accurate convective modelling procedure based on quadratic upstream interpolation. *Comput. Methods Appl. Mech. Eng.* (1979). doi:10.1016/0045-7825(79)90034-3

12. Perez, J., Menendez, M. & Losada, I. J. GOW2: A global wave hindcast for coastal applications. *Coast. Eng.* **124**, 1–11 (2017).

13. Alves, J. H. G. M. Numerical modeling of ocean swell contributions to the global wind-wave climate. *Ocean Model.* **11**, 98–122 (2006).

14. Morim, J., Hemer, M., Cartwright, N., Strauss, D. & Andutta, F. On the concordance of 21st century wind-wave climate projections. *Glob. Planet. Change* **167**, 160–171 (2018).

15. Hemer, M. A. & Trenham, C. E. Evaluation of a CMIP5 derived dynamical global wind wave climate model ensemble. *Ocean Model.* **103**, 190–203 (2016).

16. Lemos, G. *et al.* Mid-twenty-first century global wave climate projections: Results from a dynamic CMIP5 based ensemble. *Glob. Planet. Change* **172**, 69–87 (2019).

17. Hemer, M. A., Fan, Y., Mori, N., Semedo, A. & Wang, X. L. Projected changes in wave climate from a multi-model ensemble. *Nat. Clim. Chang.* **3**, 471–476 (2013).

18. Oppenheimer, M. *et al.* Sea Level Rise and Implications for Low Lying Islands, Coasts and Communities. in *IPCC Special Report on the Ocean and Cryosphere in a Changing Climate* (2019).
